# Molecular pharmacology of mGlu_7/8_ heterodimers reveals unique properties of orthosteric agonists and positive allosteric modulators

**DOI:** 10.1016/j.molpha.2026.100133

**Published:** 2026-06-06

**Authors:** Anthony S. Ferranti, Youhyun Noh, Xia Lei, Xin Lin, Joseph D. Bungard, Wesley B. Asher, Jonathan A. Javitch, Colleen M. Niswender

**Affiliations:** 1 Department of Pharmacology, Vanderbilt University, Nashville, Tennessee; 2 Warren Center for Neuroscience Drug Discovery and Vanderbilt Institute for Translational Advances, Nashville, Tennessee; 3 Departments of Psychiatry and Molecular Pharmacology and Therapeutics, Columbia University Vagelos College of Physicians and Surgeons, New York, New York; 4 Division of Molecular Therapeutics, New York State Psychiatric Institute, New York, New York; 5 Vanderbilt Kennedy Center, Vanderbilt University Medical Center, Nashville, Tennessee; 6 Vanderbilt Brain Institute, Vanderbilt University, Nashville, Tennessee; 7 Vanderbilt Institute of Chemical Biology, Vanderbilt University, Nashville, Tennessee

**Keywords:** Allosteric modulators, G protein–coupled receptors, mGlu, Central nervous system disorders

## Abstract

The metabotropic glutamate (mGlu) receptors are family C G protein–coupled receptors that are widely expressed throughout the central nervous system and function as either homodimers or heterodimers. Recent literature suggests that specific mGlu heterodimer subtypes play critical functional roles in the brain and show unique pharmacological signatures relative to homodimers. We have previously shown that mGlu_7/8_ heterodimers may modulate the induction of hippocampal synaptic plasticity using negative allosteric modulators that differentiate between mGlu_7/7_ homodimers and mGlu_7/8_ heterodimers in vitro. However, we have yet to characterize positive allosteric modulators (PAMs) targeting mGlu_7_ and mGlu_8_ in a heterodimer-specific assay. Here, we investigated the molecular pharmacology of mGlu_7/8_ heterodimers relative to mGlu_7/7_ and mGlu_8/8_ homodimers using PAMs and various orthosteric agonists using functional assays of controlled receptor activity in HEK293 cells. We demonstrate that an mGlu_8_-selective agonist is only active at mGlu_8/8_ homodimers and acts as an antagonist at mGlu_7/8_ heterodimers. In addition, we show that specific PAMs, such as VU0155094, show greater efficacy at mGlu_7/8_ heterodimers in specific assays. Overall, these findings have important implications for the physiological and behavioral roles of mGlu heterodimers and homodimers as it relates to the pharmacology of these compounds. In addition, this study provides a rationale for using a pharmacological toolkit to selectively probe mGlu_7/7_, mGlu_8/8,_ and mGlu_7/8_ dimers in native tissues.

**Significance Statement:**

Pharmacological profiling of ligands at mGlu_7/8_ heterodimers reveals that this receptor exhibits a unique pharmacological signature relative to mGlu_7/7_ and mGlu_8/8_ homodimers. This study provides an incentive to investigate the pharmacology of other mGlu heterodimers, screen for heterodimer selective compounds, and use pharmacological tools to investigate the physiological effects of mGlu_7/8_ dimers in native tissues.

## 1 Introduction

The metabotropic glutamate receptors (mGlu) are dimeric family C G protein–coupled receptors (GPCRs) that are widely expressed throughout the central nervous system (CNS).^1^ These receptors are subdivided into 3 groups comprising 8 receptor subtypes based on sequence homology and pharmacological profiles: group I (mGlu_1,5_), group II (mGlu_2,3_), and group III (mGlu_4,6,7,8_).^1^ The various mGlu receptor subtypes play unique roles in the pathophysiology of various CNS disorders and may be selectively targeted with small-molecule agonists and allosteric modulators for disease treatment.^2^^,^^3^

Emerging evidence has shown that all mGlu receptors can form heterodimers, which can dramatically affect receptor pharmacology and physiology in native tissues relative to mGlu homodimers.^4^, ^5^, ^6^, ^7^, ^8^, ^9^, ^10^, ^11^, ^12^, ^13^ Excluding mGlu_6_, which is predominantly expressed in the retina,^14^, ^15^, ^16^, ^17^, ^18^ there are 11 mGlu heterodimers that are likely to be expressed in the brain and, potentially, in the periphery.^4^ Interestingly, each subtype of mGlu heterodimer displays unique pharmacological profiles relative to homodimers as well as among various heterodimers. Emerging evidence indicates that specific mGlu heterodimer subtypes play critical physiological roles at discrete synapses in the brain.^4^^,^^6^^,^^11^^,^^12^ For example, mGlu_2/4_ heterodimers exhibit diverse expression patterns in multiple brain regions,^8^ have been shown to regulate projection-specific excitatory transmission at thalamocortical synapses in mice,^11^ and display functional expression at lateral perforant path inputs to the dentate gyrus.^9^ The unique pharmacology of mGlu heterodimers indicates that a thorough investigation of the molecular pharmacology of specific mGlu heterodimer subtypes will assist in understanding their pharmacological signatures in response to compounds that were previously deemed selective at mGlu homodimers. To this end, identifying pharmacological tools that selectively target specific mGlu heterodimers or homodimers would have utility in understanding the physiological roles of various mGlu hetero- and homodimer subtypes and assist in probing these receptors in native tissues at individual synapses and/or brain regions in which they are expressed.

We have previously demonstrated that a receptor with a pharmacological signature consistent with an mGlu_7/8_ heterodimer exists in the mouse hippocampus and modulates synaptic plasticity.^6^ More specifically, mGlu_7_ negative allosteric modulators (NAMs) that inhibit mGlu_7/8_ heterodimers block the induction of long-term potentiation (LTP) at Schaffer collateral to CA1 (SC-CA1) synapses in the hippocampus, while mGlu_7_ NAMs that do not inhibit mGlu_7/8_ heterodimers do not block SC-CA1 LTP induction.^6^ Thus, mGlu_7/8_ heterodimers may be important in modulating hippocampal-dependent learning and memory by regulating physiological processes underlying hippocampal synaptic plasticity at SC-CA1.

Numerous literature reports suggest that mutations and polymorphisms in the genes encoding for mGlu_7_ (*GRM7*) and mGlu_8_ (*GRM8*) are associated with neurodevelopmental disorders such as attention-deficit/hyperactivity disorder (ADHD) and epilepsy, among other CNS diseases.^19^, ^20^, ^21^, ^22^, ^23^, ^24^, ^25^, ^26^, ^27^, ^28^ In addition, preclinical evidence indicates that *Grm7* knockout (KO) mice exhibit seizures, cognitive impairment, ADHD-like symptoms, and neurological disorder phenotypes,^29^, ^30^, ^31^ whereas *Grm8* KO mice exhibit anxiety- and ADHD-like phenotypes.^32^, ^33^, ^34^, ^35^, ^36^, ^37^, ^38^ Furthermore, we have demonstrated that the group III mGlu receptor positive allosteric modulator (PAM) VU0422288 shows therapeutic efficacy in a Rett syndrome mouse model,^39^ and that the mGlu_7_/mGlu_8_-selective PAM VU6005649 enhances associative learning in wild-type mice.^40^ Thus, small-molecule PAMs that target the mGlu_7/8_ heterodimer may possess novel therapeutic efficacy for CNS disorders, particularly neurodevelopmental diseases associated with deficits in hippocampal neural plasticity, learning, and memory. However, the molecular pharmacology of various group III orthosteric agonists and PAMs at the mGlu_7/8_ heterodimer has yet to be thoroughly investigated. Thus, in this exploratory study, we set out to characterize the molecular pharmacology of mGlu_7/8_ heterodimers relative to mGlu_7/7_ homodimers and mGlu_8/8_ homodimers.

**Fig. 1:**
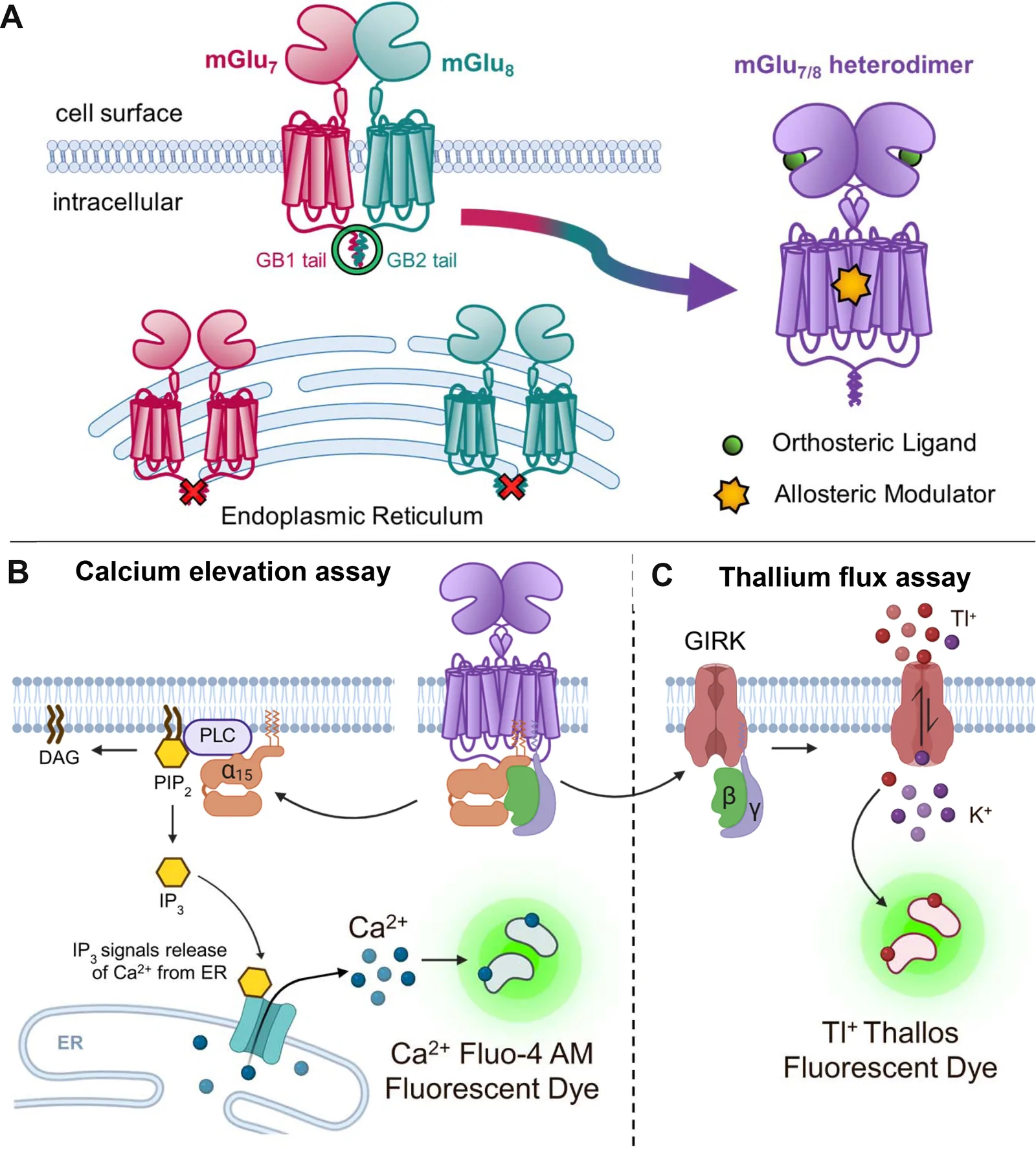
Schematic representation of the mGlu GB tails system used to assess molecular pharmacology of mGlu_7/8_ heterodimers using 2 functional assays. (A) Chimeric mGlu_7_ and mGlu_8_ constructs with modified C-termini containing either GABA_B1_ (GB1) or GABA_B2_ (GB2) receptor tails were transfected into human embryonic kidney 293A (HEK293A) cells to allow for controlled dimer subunit composition at the cell surface. Both GB1 and GB2 tails contain an endoplasmic reticulum retention signal that, only when constructs containing GB1 and GB2 are transfected together, is masked, permitting only GB1/GB2 receptor combinations to be expressed on the cell surface. In this example, mGlu_7_ GB1 is cotransfected with mGlu_8_ GB2, leading to restricted expression and pharmacological assessment of mGlu_7/8_ heterodimers. (B) HEK293 cells stably express either the promiscuous G protein G_α15_ to allow group III mGlu receptors to couple to calcium elevation or (C) the G protein–coupled inwardly rectifying potassium channels (GIRKs) that reflect group III mGlu receptor signaling through native G_i/o_-coupled G proteins. Both methods were used to assess the downstream functional effects of mGlu receptor activation using orthosteric ligands and allosteric modulators. DAG, diacylglycerol; ER, endoplasmic reticulum; mGlu, metabotropic glutamate receptors; PIP2, phosphatidylinositol 4,5-bisphosphate; PLC, phospholipase C.

Using chimeric mGlu_7_ and mGlu_8_ constructs modified to control for mGlu dimer subunit composition at the cell surface (Fig. 1A, adapted from Jean-Philippe Pin Laboratory, University of Montpellier),^41^, ^42^, ^43^ we assessed the molecular pharmacology of mGlu_7/8_ heterodimers relative to mGlu_7/7_ and mGlu_8/8_ homodimers using various group III orthosteric agonists and PAMs in 2 functional assays of receptor activity: a calcium elevation assay using the promiscuous G protein G_α15_ (Fig. 1B) and a G protein–coupled inwardly rectifying potassium (GIRK) channel thallium flux assay (Fig. 1C). In some experiments, we also used a complemented donor acceptor resonance energy transfer (CODA-RET) assay to selectively measure G protein recruitment by mGlu_7/8_ heterodimers without contribution of signaling from homodimers expressed in the same cells.^6^^,^^44^^,^^45^ We reveal that, relative to homodimers, mGlu_7/8_ heterodimers show unique agonist selectivity and enhanced PAM efficacy pharmacology in response to specific allosteric modulators.

## 2 Materials and methods

### 2.1 Drugs

The group III mGlu receptor agonists DL-2-Amino-4-phosphonobutyric acid (DL-AP4) (cat. 0101), L-(+)-2-Amino-4-phosphonobutyric acid (L-AP4) (cat. 0103), L-glutamic acid (glutamate) (cat. 0218), and (*S*)-3,4-dicarboxyphenylglycine ((*S*)-DCPG) (cat. 1302) were purchased from Tocris. The group III mGlu receptor PAMs (VU0155094, VU0422288, VU6027459, VU6046980, and VU6046981) were synthesized in house as previously described.^46^, ^47^, ^48^ VU6005649^40^ was purchased from MedChemExpress (cat. HY-107982).

### 2.2 DNA construction

Plasmids for mGlu receptors were constructed with GABA_B1_ (GB1) and GABA_B2_ (GB2) tails as previously described.^41^, ^42^, ^43^^,^^49^^,^^50^ Hemagglutinin-tagged rat mGlu_7_ GB1 and rat mGlu_7_ GB2 plasmids were constructed as indicated in the article by Lei et al.^50^ Rat mGlu_8_ GB1 and rat mGlu_8_ GB2 plasmids were constructed with a c-Myc (MYC) tag sequence inserted directly after the signal peptide in the mGlu_8_ N-terminus. The C-terminus of the rat mGlu_8_ receptor was replaced after I870 with 3 alanines, followed by the coiled-coiled domain of the GB1 tail (amino acids 871–925) or GB2 tail (amino acids 871–927) as previously described in the article by Huang et al^43^ and then a KKTN amino acid sequence encoding for an endoplasmic reticulum retention signal^51^ was included as the 4 final amino acids. The entire sequence for either rat mGlu_8_ GB1 or rat mGlu_8_ GB2 was fused into a pcDNA3 vector via EcoRI and NotI enzyme restriction sites. The constructed rat mGlu_8_ GB1 and rat mGlu_8_ GB2 plasmids were sequenced at Azenta/GENEWIZ via Plasmid-EZ sequencing to confirm the full-length plasmid sequences and read counts. Listed below are the C-termini 1-letter amino acid sequences for each mGlu_8_ GB tail construct:

1. *mGlu8 GB1*: AAATGSSTNNNEEEKSRLLEKENRELEKIIAEKEERVSELRHQLQSRQQLKKTN∗
2. *mGlu8 GB2*: AAATSTSVTSVNQASTSRLEGLQSENHRLRMKITELDKDLEEVTMQLQDTPEKKTN∗

### 2.3 Cell lines and cell culture

Human embryonic kidney 293A (HEK293A) cells were purchased from the American Tissue Culture Collection. These cells were stably transfected with G_*α*15_, a promiscuous G protein, to promote calcium elevation on coupling to G_i/o_-coupled receptors as previously described.^46^^,^^52^^,^^53^ In addition, HEK293 cells stably expressing GIRK channels 1 and 2 were used to assess mGlu receptor pharmacology related to G_i/o_/G_*β*γ_-mediated signaling mechanisms.^50^^,^^54^ Cells were passed in growth media consisting of Dulbecco’s modified Eagle’s medium (DMEM; cat. 11960-044; Thermo Fisher), supplemented with 10% fetal bovine serum (FBS; cat. A5670701; Thermo Fisher), 20 mM HEPES (cat. 15630-080; Thermo Fisher), 1 mM Na pyruvate (cat. 11360-070; Thermo Fisher), 2 mM GlutaMAX (cat. 35050-061; Thermo Fisher), 0.1 mM nonessential amino acids (cat. 11140-050; Thermo Fisher), 1× antibiotic/antimycotic (cat. 15240-062; Thermo Fisher) under G418 sulfate (700 *μ*g/mL) (cat. 10131-027; Thermo Fisher) selection to maintain stable expression. All cell culture reagents were purchased from Invitrogen (Thermo Fisher Scientific). Cells were routinely monitored every 2–3 months for mycoplasma contamination using a LookOut Mycoplasma PCR Detection Kit (Sigma-Aldrich). The cells for the studies described here were not mycoplasma positive.

### 2.4 Transient transfections

Transient transfections were performed using the Lipofectamine 3000 protocol according to the manufacturer’s instructions (cat. L3000-008; Invitrogen). Lipofectamine 3000 reagent and DNA (2 *μ*g/plasmid) were first diluted in Opti-MEM transfection media (cat. 31985-070; Thermo Fisher) and then subsequently combined with P3000 reagent diluted in Opti-MEM to form a DNA-lipid complex. Each DNA/Lipofectamine 3000/P3000 mixture complex was vortexed for 15 seconds, followed by a 10–15-minute incubation at room temperature. After the incubation step, each complex was added dropwise to HEK293 G_α15_ cells at ∼50%–70% confluency in a 10-cm tissue culture–treated dish (cat. 353003; Corning Inc). Lipofectamine/DNA complexes were incubated with HEK293 G_*α*15_ cells in Opti-MEM media in 10-cm dishes for 24 hours.

### 2.5 Generation of polyclonal stable cell lines

To generate HEK293A cell lines stably expressing mGlu GB tail constructs, we used restriction cloning to generate 3 plasmids that each express a unique antibiotic resistance gene. A total of 4 stable cell lines were generated for each mGlu_7_/mGlu_8_ dimer condition: (1) mGlu_7/7_ homodimer (mGlu_7_ GB1 + mGlu_7_ GB2), (2) mGlu_8/8_ homodimer (mGlu_8_ GB1 + mGlu_8_ GB2), (3) mGlu_8/7_ heterodimer (mGlu_8_ GB1 + mGlu_7_ GB2), and (4) mGlu_7/8_ heterodimer (mGlu_7_ GB1 + mGlu_8_ GB2). Cells were transfected with Lipofectamine 3000 following the procedure described for transient transfections. After 24 hours, growth media with antibiotic selector was applied to cells and changed every 48 hours to induce selective pressure. HEK293A polyclonal cell lines were transfected with mouse G_*α*15_ protein in a pIREShyg3 vector expressing a hygromycin antibiotic resistance gene. Once steady cell growth and stable plasmid expression were achieved, mGlu-GB2–tailed constructs in a pcDNA3 vector expressing the Geneticin/G418 sulfate resistance gene were transfected into the pIREShyg3 G_α15_ cell lines. After steady growth and plasmid expression in these cell lines were achieved, mGlu-GB1–tailed constructs in a pIRESpuro3 vector expressing the puromycin resistance gene were transfected. Kill curves were generated for each antibiotic before transfections to determine the appropriate concentration of antibiotic. Cells were passed in growth media containing the antibiotic selectors Geneticin/G418 sulfate (700 *μ*g/mL) (Gibco cat. 10131-027), hygromycin (100 *μ*g/mL) (Invitrogen cat. 10687010), and puromycin (250 ng/mL) (Sigma cat. P8833) to maintain stable expression of G_α15_ and each mGlu-GB-tailed construct. After 2–3 weeks, polyclonal stable cell lines showed steady growth with minimal cell death, and experiments were subsequently used 4 weeks after the last transfection.

### 2.6 Calcium elevation assay

Transfected HEK293A G_α15_ cells were trypsinized for 5 minutes and resuspended in assay media (DMEM containing 10% dialyzed FBS, 20 mM HEPES, 100 units/mL penicillin/streptomycin, and 1 mM sodium pyruvate). For each dimer condition, mGlu_7_- and mGlu_8_-GB-tailed constructs fused to the pcDNA3 vector were cotransfected to generate the following conditions: (1) mGlu_7/7_ homodimer (mGlu_7_ GB1 + mGlu_7_ GB2), (2) mGlu_8/8_ homodimer (mGlu_8_ GB1 + mGlu_8_ GB2), (3) mGlu_8/7_ heterodimer (mGlu_8_ GB1 + mGlu_7_ GB2), and (4) mGlu_7/8_ heterodimer (mGlu_7_ GB1 + mGlu_8_ GB2). Resuspended cells were plated into black-walled, clear-bottomed, poly-d-lysine–coated 384-well assay plates (cat. 356663; Corning Inc) at a density of 20,000 cells/well (20 *μ*L/well) and incubated overnight at 37 °C in the presence of 5% CO_2_. The position of each condition was varied on the plate to counterbalance conditions across experimental days. The next day, assay media was removed, and the cells were washed 3 times with assay buffer (1× Hank’s Balanced Salt Solution, 20 mM HEPES, 4.17 mM sodium bicarbonate [cat. S6014, Sigma], and 2.5 mM probenecid [cat. P8761, Sigma] in Milli-Q H_2_O) using an ELX plate washer. Probenecid was dissolved in 1N NaOH and directly added to assay buffer on the day of each experiment. After plate washes, cells were immediately loaded with 1 *μ*M (20 *μ*L/well) of Fluo-4, AM Loading Dye (cat. F14202) (Thermo Fisher) using a Thermo Fisher Combi (Thermo Fisher) and incubated at 37 °C for 50 minutes. Loading dye was prepared as a 2.3 mM stock in DMSO and mixed in a 1:1 ratio with 10% (w/v) pluronic acid F-127 and diluted in assay buffer. The loading dye was removed after 50 minutes, and cells were washed again 3 times with assay buffer, and 20 *μ*L of assay buffer was subsequently added to each well. Cells were then incubated with assay buffer for 10 minutes at room temperature. Allosteric modulators were dissolved in DMSO to achieve a 10 mM stock concentration and then diluted in assay buffer on the day of experiments. For the vehicle group in experiments with allosteric modulators, assay buffer with 0.1% (v/v) DMSO was used. For Schild experiments with 2 agonists, assay buffer was used as vehicle. Compound and vehicle were then transferred to 384-well polypropylene-coated clear plates (cat. 264573; Thermo Fisher) at 2× final concentration and then diluted in assay buffer to achieve final concentrations of 10 *μ*M for compounds and 0.1% (v/v) DMSO for vehicle. Agonists (glutamate DL-AP4 and (*S*)-DCPG) were initially dissolved in 0.1M NaOH or H_2_O to achieve a concentration of 100 mM; pH of agonist solutions was adjusted to 7.2 using 6N HCl, and 100 mM aliquots of each agonist were stored at –20 °C and thawed on the day of each experiment. Agonists were serially diluted by hand or using a Bravo Velocity 11 (Agilent Technologies) in assay buffer at 5× final concentrations to generate 11-point concentration-response curves. Calcium flux was measured using a Functional Drug Screening System (FDSS/*μ*Cell; Hamamatsu Photonics). During the assay, readings were taken at 1 Hz (excitation, 470 ± 20 nm; emission, 540 ± 30 nm) to establish a fluorescence baseline. Twenty microliters (2×) of vehicle or a constant concentration of compound was added to the cells, and the response was measured. Individual raw data points were then generated at each second during the assay by dividing the fluorescence at time 0 to generate a ratio. For each concentration tested, the peak fluorescence value from the kinetic raw traces was calculated by subtracting the minimum fluorescence value (at 131–140 seconds) from the maximum fluorescence value (at 142–268 seconds). This peak fluorescence value was then converted to a percent maximum value based on single saturating concentration of agonist (DL-AP4) at mGlu_8/8_ homodimers equal to 100%. The percent maximum value at each concentration tested was then plotted and analyzed for each concentration-response curve (CRC).

### 2.7 GIRK-mediated thallium flux assay

HEK293A cells stably expressing GIRK 1/2 channels were transiently cotransfected with mGlu_7_- and mGlu_8_-GB-tailed constructs fused to pcDNA3 vector to generate a total of 4 conditions for each mGlu_7_/mGlu_8_ dimer pair: mGlu_7/7_ homodimer (mGlu_7_ GB1 + mGlu_7_ GB2), mGlu_8/8_ homodimer (mGlu_8_ GB1 + mGlu_8_ GB2), mGlu_7/8_ heterodimer (mGlu_7_ GB1 + mGlu_8_ GB2), and mGlu_8/7_ heterodimer (mGlu_8_ GB1 + mGlu_7_ GB2). Cells were grown at 37 °C in the presence of 5% CO_2_ in growth media under G418 sulfate (700 *μ*g/mL) selection to maintain stable expression. Transfected HEK293 GIRK cells were harvested by treating with trypsin (cat. no. 12605-010; Thermo Fisher) for 5 minutes and resuspended in assay media (DMEM containing 10% dialyzed FBS, 20 mM HEPES, and 1 mM sodium pyruvate). Cells were plated at a density of 15,000 cells per well (20 *μ*L/well) into black-wall, clear-bottom, poly-d-lysine–coated 384-well assay plates (cat. no. 354663; Corning) and incubated overnight at 37 °C in the presence of 5% CO_2_. The 4 transfection conditions were plated in separate horizontal sections of the plate, with their positions rotated across assay runs to counterbalance potential positional variability. The following day, assay media was removed, and the cells were washed 3 times with assay buffer (1× Hank’s Balanced Salt Solution, 20 mM HEPES, and 4.17 mM sodium bicarbonate) using an ELX plate washer. Immediately after washing, cells were loaded with 1*μ*M (20 *μ*L/well) of Thallos AM green fluorescent thallium (Tl) indicator (cat. no. 1381; ION Biosciences) using a Combi dispenser (Thermo Fisher). Thallos dye was prepared as a 2.85 mM stock in DMSO mixed 1:1 with 10% (w/v) pluronic acid F-127 and diluted in assay buffer before use. Cells were incubated with the dye at room temperature for 60 minutes in the dark and then washed 3 additional times with assay buffer. A final volume of 20 *μ*L assay buffer was added to each well, and cells were incubated for 10 minutes at room temperature before initiating the thallium flux assay.

Test compound VU0155094 was dissolved in DMSO to a stock concentration of 10 mM. Assay buffer containing 0.1% (v/v) DMSO was used as the vehicle control. Both compound and vehicle were diluted to 2× final concentration in assay buffer and dispensed into polypropylene-coated clear 384-well plates (cat. no. 264573; Corning). Agonists (glutamate, DL-AP4, and (*S*)-DCPG) were serially diluted in thallium buffer (125 mM sodium bicarbonate, 1 mM magnesium sulfate, 1.8 mM calcium sulfate, 5 mM glucose, 12 mM thallium sulfate, and 10 mM HEPES) using a Bravo Velocity 11 (Agilent Technologies). Serial dilutions were prepared at 5× final concentration to generate either 10-point (glutamate and (*S*)-DCPG) or 11-point (DL-AP4) CRCs. Thallium flux was measured using the FDSS *μ*Cell kinetic plate reader (Hamamatsu Photonics). Fluorescence was recorded at 1 Hz (excitation: 480 ± 20 nm; emission: 540 ± 30 nm) to establish a baseline. After baseline acquisition, 20 *μ*L of either compound or vehicle (2×) was added to the wells, and fluorescence was recorded after each second. At 141 seconds, agonist or vehicle (thallium buffer) was added, and fluorescence was monitored for an additional 156 seconds. Raw fluorescence values were normalized by dividing each time point by the signal at time zero to generate fluorescence ratio traces for analysis. To measure functional responses, a slope was calculated to reflect the rate at which the cells flux thallium in the presence of agonist compared with vehicle alone. This slope was calculated from 147 to 157 seconds using the Microsoft Excel SLOPE (known_y’s, known_x’s) function, where fluorescence values were “known_y” values and seconds were “known_x” values. The average slope of all vehicle wells per condition was subtracted from wells containing agonist to generate a normalized slope value for each concentration point on the plate. Normalized slope values were then converted to a percent agonist maximum value normalized to 100%. This normalized percent maximum value was based on the mGlu_8/8_ homodimer slope in response to 1 mM DL-AP4 run in each experiment on the same plate. The data were then plotted in GraphPad Prism (version 10 and greater), and CRCs were fit using either a 4-parameter or 3-parameter (log)agonist versus response logistical equation depending on which model was calculated to have the best-fit.

### 2.8 Complemented donor acceptor resonance energy transfer assay

For construction and transfection of expression vectors, cDNAs for rat mGlu_7_ and mGlu_8_ were N-terminally tagged with a hemagglutinin epitope tag using standard molecular biology procedures. cDNAs encoding the split fragments of Nano luciferase (NanoLuc; Promega), LgBit (residues 1–158), or HiBit (residues 159–169: VSGWRLFKKIS) were fused in frame to the C-terminus of related mGlu receptor sequences mGlu_7_ and mGlu_8_ following the linker “EFPPARATLPPARATLE” in the pcDNA3.1 vector. Mini-G_αo_ fused to mVenus, mVenus-miniG_αo_, was a gift from Dr Nevin Lambert (Augusta University).^55^ The excitatory amino acid transporter 3 in the pcDNA3.1 was a gift from Dr Joshua Levitz (Weill Cornell Medicine). All constructs were confirmed with sequencing analysis. CODA-RET assays were performed in 293T cells (American Type Culture Collection, CRL-3216) from embryonic kidney tissue of Homo sapiens (HEK293T). The cells were maintained in DMEM, high glucose (Gibco) supplemented with 10% fetal bovine serum (Corning Inc) and 1% penicillin-streptomycin (Corning Inc) at 37 °C with 5% CO_2_. Before transfection, HEK293T cells were grown to ∼70% confluency in 10-cm tissue culture dishes. The pcDNA 3.1 (+) plasmids encoding mGlu_7_ or mGlu_8_ protomers (mGlu_7_ homodimer expression: 100 ng mGlu_7_-LgBit and 100 ng mGlu_7_-HiBit; mGlu_8_ homodimer expression: 50 ng mGlu_8_-LgBit and 50 ng mGlu_8_-HiBit; mGlu_7/8_ heterodimer expression: 200 ng mGlu_7_-LgBit and 100 ng mGlu_8_-HiBit) were cotransfected with 5 *μ*g mVenus-miniGαo and 1 *μ*g excitatory amino acid transporter 3 into HEK293T cells cultured in a 10-cm dish using polyethylenimine hydrochloride (PEI) Max 4000 (cat. 49553-93-7; Polysciences Inc) at a ratio of 9 *μ*g PEI Max per 1 *μ*g total plasmid. Transfected cells were incubated at 37 °C with 5% CO_2_ for 48 hours. Before experiments, the cells were incubated with DMEM, high glucose, GlutaMAX supplement, pyruvate (Gibco) in the absence of fetal bovine serum for 1 hour at 37 °C with 5% CO_2_. After expression, the cells were dissociated from the 10-cm plate using enzyme-free dissociation solution (Millipore-Sigma). After dissociation, the cells were collected and spun at 1000*g* for 5 minutes. The supernatant was discarded, and the cells were washed twice with prewarmed Dulbecco’s phosphate-buffer saline. Approximately 150,000 cells per well were distributed in black frame, white well 96-well plates (PerkinElmer). Furimazine (5 *μ*M), the substrate for NanoLuc, was added to each well and immediately after the addition of furimazine, Dulbecco’s phosphate-buffer saline containing the indicated mGlu receptor agonist or vehicle was loaded to each well. Five minutes after ligand stimulation at 37 °C, the fluorescence and luminescence signals were quantified using a Pherastar FS plate reader (BMG Labtech). The bioluminescence resonance energy transfer ratio was determined by calculating the ratio of the mVenus signal (535 nm) to the NanoLuc signal (485 nm).

### 2.9 Data analysis and statistics

For calcium and thallium flux experiments, data were analyzed as described in the article by Rodriguez et al.^56^ For transient transfections, the data reported were normalized to the percent maximal DL-AP4 plus vehicle response of the mGlu_8_ GB1/GB2 homodimer condition. The agonist pEC_50_ and maximal response in the presence of vehicle (DMSO) or PAM (10 *μ*M) were determined using a 3-parameter or 4-parameter logistical equation (depending on best-fit values) in GraphPad Prism. Each experiment was performed with 3 replicates per day (all replicates averaged into 1 *N* value per day), and each experiment was then performed 3 separate times on individual days to achieve a total *N* = 3. For all experiments, each separate heterodimer condition was run on the same plate to control for potential differences between each condition. During post hoc analysis, heterodimer responses were combined because there were no differences in efficacy and potency detected between heterodimer configurations (mGlu_7_ GB1 + mGlu_8_ GB2 vs mGlu_7_ GB2 + mGlu_8_ GB1) (Supplemental Fig. 1). For fold-shift experiments comparing PAMs across dimer conditions, data were normalized to a percent maximum glutamate within each condition (1 mM for mGlu_8/8_ and mGlu_7/8_ conditions; 5 mM for mGlu_7/7_). For experiments examining (*S*)-DCPG as a competitive antagonist at the mGlu_7/8_ heterodimer receptor, CRCs were fit using the Gaddum/Schild EC_50_ shift function in GraphPad Prism version 10 and higher. For the Schild regression analysis, the dose ratio (DR) was calculated by dividing the pEC_50_ concentration of agonist (DL-AP4) alone in the vehicle condition by the pEC_50_ concentration of agonist with antagonist ((*S*)-DCPG) present. The Log of the DR minus 1 (Log(DR – 1)) was plotted against the log concentration of (*S*)-DCPG for each point, and a simple linear regression analysis was performed in GraphPad Prism. Log(DR – 1) values that were less than 0 were excluded from analysis. Data from the CODA-RET assays were plotted and fit using GraphPad Prism version 10 or higher (GraphPad Software). The dose-response curves were fitted by nonlinear regression to the log (agonist) versus response model with a standard Hill slope equal to 1.

In all figures, data and error bars represent mean ± SD. This study was designed to be exploratory in nature; thus, all statistical analyses and *P* values reported should not be interpreted as hypothesis testing but rather as merely descriptive to help the reader’s interpretation of the study. In cases where 2 experimental variables were being compared, a 2-tailed unpaired *t* test was performed to compare the mean differences between the 2 groups. For experiments with more than 2 experimental variables, a 1-way ANOVA was performed, followed by a Tukey’s post hoc test to detect for differences within specific groups. For experiments with multiple categorical variables, a 2-way ANOVA was performed, followed by a Tukey’s post hoc test. All statistical tests were run with 95% CIs.

## 3 Results

### 3.1 Controlled mGlu dimer subunit composition at the cell surface allows for the assessment of the molecular pharmacology of mGlu_7/8_ heterodimers

To specifically test the effects of ligands at controlled homodimer or heterodimer combinations, we generated chimeric mGlu_7_ and mGlu_8_ receptor constructs with modified C-termini containing GABAB1 (GB1) and GABAB2 (GB2) receptor tails (GB tails) as previously described.^41^, ^42^, ^43^^,^^50^ These GB tails each contain an endoplasmic reticulum retention signal that restricts receptor expression to the endoplasmic reticulum when expressed alone. When GB1- and GB2-containing receptors are coexpressed together in HEK293 cells, only GB1/GB2 receptor dimer expression occurs at the cell surface (Fig. 1A). This allowed us to analyze the molecular pharmacology of mGlu_7_/mGlu_8_ dimer combinations using a calcium elevation assay (Fig. 1B) and a GIRK channel functional assay (Fig. 1C) of receptor efficacy.^54^

Using the GB-tailed constructs, 4 possible mGlu dimer combinations were expressed in HEK293 cells and analyzed: (1) mGlu_7/7_ homodimer (mGlu_7_ GB1 + mGlu_7_ GB2), (2) mGlu_8/8_ homodimer (mGlu_8_ GB1 + mGlu_8_ GB2), (3) mGlu_8/7_ heterodimer (mGlu_8_ GB1 + mGlu_7_ GB2), and (4) mGlu_7/8_ heterodimer (mGlu_7_ GB1 + mGlu_8_ GB2). Control conditions were also generated by transfecting 1 mGlu-GB-tailed construct with an empty vector (eg, pcDNA3 + mGlu_8_ GB2) or 2 mGlu constructs with the same GB tail in the calcium elevation assay (eg, mGlu_7_ GB2 + mGlu_8_ GB2) (Supplemental Fig. 2). These experiments demonstrated that the mGlu_8_ GB2 plasmid showed consistent “leak” that resulted in ∼20% functional activity compared with the mGlu_8/8_ homodimer condition (Supplemental Fig. 2A), whereas all other plasmid conditions (mGlu_8_ GB1, mGlu_7_ GB1, and mGlu_7_ GB2) showed no response (Supplemental Fig. 2B). However, the observation that the mGlu_7_ GB2 condition does not exhibit “leak” may be because mGlu_8_ shows greater sensitivity to the agonist DL-AP4 relative to mGlu_7_. Therefore, the mGlu_7_ GB2 condition may “leak” to a similar extent to that of mGlu_8_ GB2, and it is possible that this assay is not sensitive in detecting it.

### 3.2 The mGlu_7/8_ heterodimer exhibits unique pharmacology and selectivity profiles relative to homodimers in response to group III agonists

We assessed the pharmacology of mGlu_7/7_ homodimers, mGlu_8/8_ homodimers, and mGlu_8/7_ and mGlu_7/8_ heterodimer conditions by generating CRCs using glutamate, the group III mGlu agonist DL-AP4, and the selective mGlu_8_ agonist (*S*)-DCPG^57^ in the calcium elevation assay. Functional responses for each dimer were normalized to a percent maximal DL-AP4 (5 mM) response for the mGlu_8/8_ homodimer condition to compare relative efficacies among homo and heterodimers. Importantly, the 2 heterodimer configurations did not differ in response at any of the concentrations tested for glutamate (Supplemental Fig. 1A, 2-way ANOVA; no main effect of heterodimer configuration *F*(1,24) = 3.181, *P* = .0871; main effect of concentration *F*(5,24) = 8.665, *P* < .0001; no interaction effect *F*(5,24) = 0.1114, *P* = .9887), and no differences in glutamate potency were detected between heterodimer configurations (Supplemental Fig. 1B, unpaired *t* test; *t*(4) = 0.4422, *P* = .6812). Similarly, no efficacy differences between heterodimers were detected among any concentration of DL-AP4 tested (Supplemental Fig. 1C, 2-way ANOVA; no main effect of heterodimer configuration *F*(1,28) = 0.2082, *P* = .6517; main effect of concentration *F*(6,28) = 16.74, *P* < .0001; no interaction effect *F*(6,28) = 0.07543, *P* = .07543), and no DL-AP4 potency differences were observed between heterodimers (Supplemental Fig. 1D, unpaired *t* test; *t*(4) = 0.4770, *P* = .6583). Therefore, heterodimer responses were subsequently combined during data analysis by averaging the raw data from each heterodimer configuration replicate on individual assay days.

**Fig. 2:**
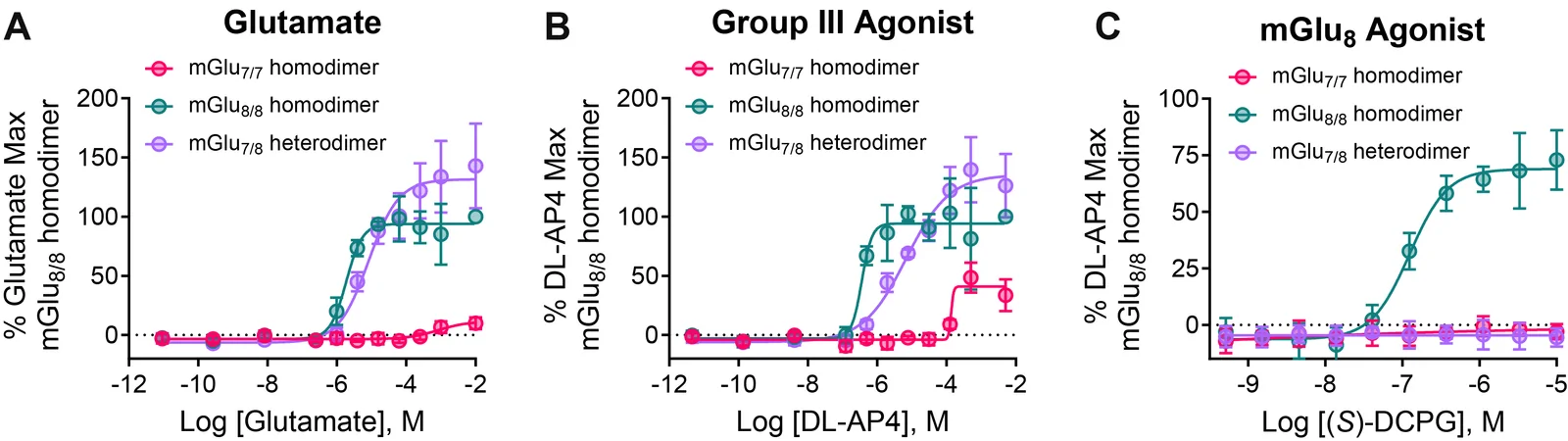
Orthosteric agonists reveal unique pharmacology of mGlu_7/8_ heterodimers. (A) Glutamate concentration-response curves (CRCs) show unique pharmacology of mGlu_7/8_ heterodimers (purple) relative to the mGlu_7/7_ (red) and mGlu_8/8_ homodimer (teal) conditions. The glutamate pEC_50_ was 5.73 ± 0.10 (mean pEC_50_ ± SD) for mGlu_8/8_ homodimers and 5.05 ± 0.14 for mGlu_7/8_ heterodimers. pEC_50_ values were not calculated for mGlu_7/7_ homodimers due to low functional activity of this receptor in response to glutamate. (B) CRCs plotted for the group III agonist DL-AP4 show that mGlu_7/8_ heterodimers (purple) exhibit differential potency relative to the mGlu_7/7_ (red) and mGlu_8/8_ homodimer (teal) conditions. For each dimer, the pEC_50_ was 3.86 ± 0.01 (mean pEC_50_ ± SD) for mGlu_7/7_ homodimers, 6.41 ± 0.06 for mGlu_8/8_ homodimers, and 5.17 ± 0.20 for mGlu_7/8_ heterodimers. (C) The mGlu_8_ selective agonist (*S*)-3,4-dicarboxyphenylglycine ((*S*)-DCPG) is unable to activate mGlu_7/8_ heterodimers, suggesting that this receptor likely requires both agonist binding sites to be occupied for saturable activation of the receptor. Data in panel (A) are normalized to percent maximum activation of the mGlu_8/8_ homodimer in response to glutamate; data in panels (B) and (C) are normalized to percent maximum activation of the mGlu_8/8_ homodimer in response to DL-AP4 assayed on the same plate. *N* = 3 independent experiments; averaged triplicates obtained on separate days. Data plotted are mean ± SD. DL-AP4, DL-2-amino-4-phosphonobutyric acid; mGlu, metabotropic glutamate receptors.

When generating glutamate CRCs (Fig. 2A), the glutamate pEC_50_ was 5.73 ± 0.10 (mean pEC_50_ ± SD) for mGlu_8/8_ homodimers and 5.05 ± 0.14 for mGlu_7/8_ heterodimers. Because of the low functional efficacy of glutamate at mGlu_7/7_ homodimers in this assay, pEC_50_ values were not calculated for this receptor. mGlu_7/8_ heterodimers exhibited a significantly right-shifted glutamate potency relative to mGlu_8/8_ homodimers (unpaired *t* test of receptor pEC_50_; *t*(4) = 6.65, ∗∗*P* < .01). In response to DL-AP4 (Fig. 2B), the pEC_50_ was 3.86 ± 0.01 (mean pEC_50_ ± SD) for mGlu_7/7_ homodimers, 6.41 ± 0.06 *μ*M for mGlu_8/8_ homodimers, and 5.17 ± 0.20 for mGlu_7/8_ heterodimers. The potency values for each receptor were all different from one another, indicating that mGlu_7/7_ homodimers, mGlu_8/8_ homodimers, and mGlu_7/8_ heterodimers all exhibit differential responses to DL-AP4 (ordinary 1-way ANOVA; *F*(2,6) = 333.2, ∗∗∗∗*P* < .0001; Tukey’s post hoc for mGlu_7/7_ vs mGlu_7/8_ ∗∗*P* < .0001, mGlu_8/8_ vs mGlu_7/8_ ∗∗∗∗*P* < .0001, mGlu_7/7_ vs mGlu_8/8_ ∗∗∗∗*P* < .0001). Interestingly, when fitting both glutamate and DL-AP4 CRCs to a 4-parameter curve fit, mGlu_7/8_ heterodimers exhibited a Hill slope value of less than 1 (glutamate Hill slope value = 0.82; DL-AP4 Hill slope value = 0.70), consistent with the idea that the heterodimer possess 2 nonequivalent orthosteric binding sites for the same ligand.^58^ Strikingly, CRCs with (*S*)-DCPG (Fig. 2C) revealed that there was no response from the mGlu_7/8_ heterodimer, indicating that this compound is functionally selective for mGlu_8/8_ homodimers among the receptor subtypes tested in this assay. Taken together, the shallow agonist Hill slopes and inactivity of (*S*)-DCPG at the mGlu_7/8_ heterodimer are consistent with the mGlu_7/8_ heterodimer exhibiting unique activation mechanisms relative to when mGlu_7_ and mGlu_8_ are in homodimer configurations.

### 3.3 (S)-DCPG acts as a selective antagonist at the mGlu_7/8_ heterodimer by competing with DL-AP4 at the mGlu_8_ protomer of the dimer

**Fig. 3:**
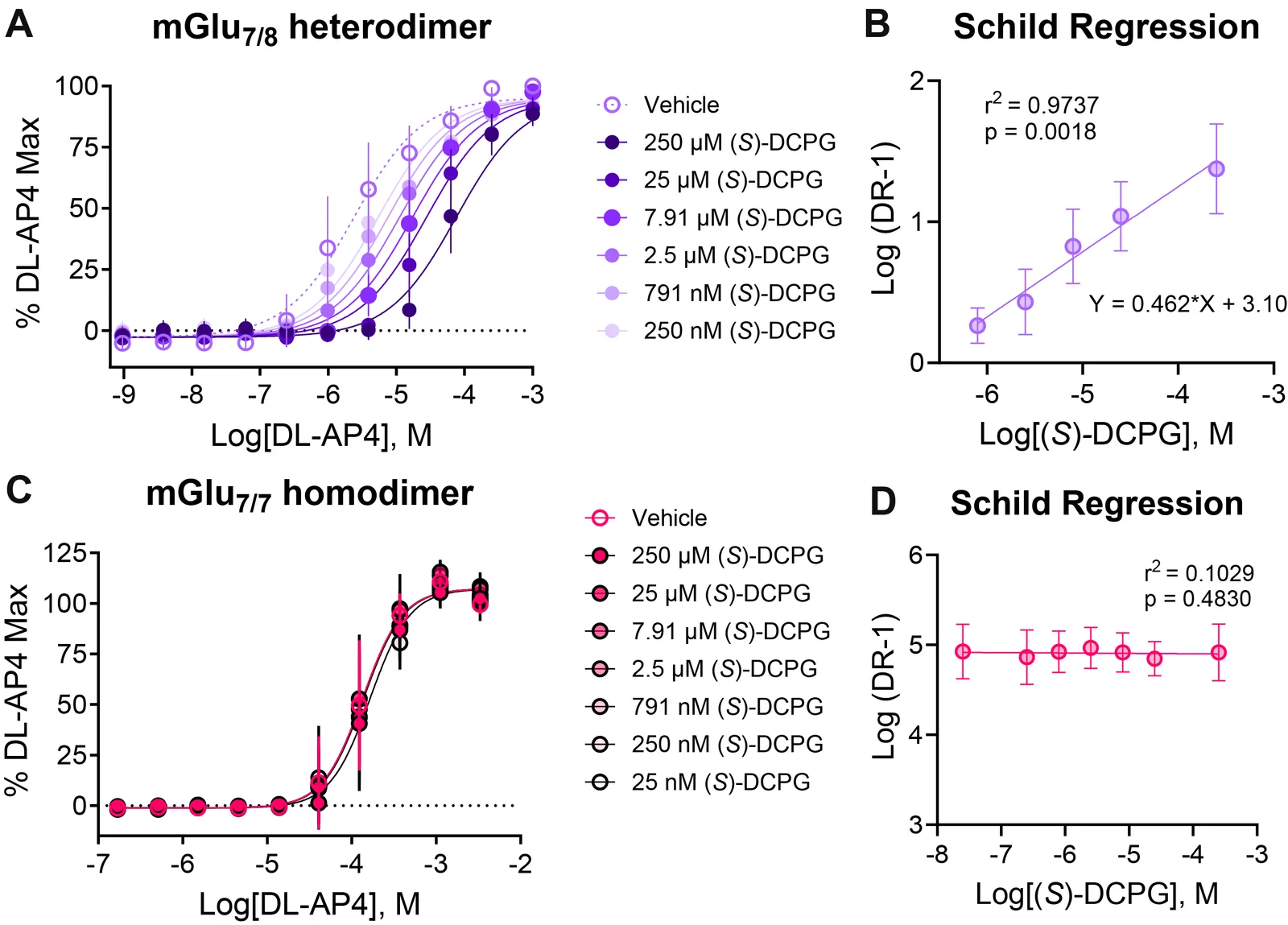
The mGlu_8_ agonist (*S*)-DCPG acts as a competitive antagonist at mGlu_7/8_ heterodimers. (A) DL-AP4 CRCs show that, with increasing concentrations of (*S*)-DCPG, the potency of DL-AP4 is progressively reduced at mGlu_7/8_ heterodimers. Importantly, the maximal efficacy of mGlu_7/8_ heterodimers in response to a saturating concertation of DL-AP4 (1 mM) is not significantly reduced at any concentration of (*S*)-DCPG applied, suggesting a competitive interaction (1-way ANOVA measuring differences in max efficacy of DL-AP4 in the presence of varying concentrations of (*S*)-DCPG; *F*(7,16) = 1.864, *P* = .1432). (B) Schild regression analysis revealed a significant correlation between the concentration of (*S*)-DCPG and dose ratio (DR) (simple linear regression analysis; *r*^2^ = 0.9737, *P* < .01; slope equation: Y = 0.4616x + 3.096). (C) When tested at the mGlu_7/7_ homodimer, (*S*)-DCPG does not show antagonist activity in a Schild regression experiment, as indicated in (D) by the lack of correlation between the concentration of (*S*)-DCPG and DR (simple linear regression analysis; *r*^2^ = 0.1029, *P* = .483; slope equation: Y = –0.0041x + 4.885). CRC, concentration-response curve; DL-AP4, DL-2-amino-4-phosphonobutyric acid; mGlu, metabotropic glutamate receptors; (S)-DCPG, (*S*)-3,4-dicarboxyphenylglycine.

As we found that (*S*)-DCPG was unable to activate the mGlu_7/8_ heterodimer, we hypothesized that (*S*)-DCPG may act as a selective competitive antagonist at the mGlu_7/8_ heterodimer by blocking the binding and efficacy of DL-AP4 through the mGlu_8_ protomer of the dimer. To test this hypothesis, we generated DL-AP4 CRCs after pretreating mGlu_7/8_ heterodimer-expressing polyclonal stable cell lines with increasing concentrations of (*S*)-DCPG and performed a Schild regression analysis (Fig. 3). In support of our hypothesis, we observed a linear rightward shift in DL-AP4 potency with increasing concentrations of (*S*)-DCPG without affecting the maximal efficacy of DL-AP4, which is consistent with (*S*)-DCPG acting as a competitive antagonist at the heterodimer (Fig. 3A). A Gaddum/Schild curve fit resulted in a linear Schild regression for the mGlu_7/8_ heterodimer using simple linear regression analysis; *r*^2^ = 0.9737, *F*(1,3) = 111.1; *P* = .0018 (Fig. 3B). Notably, the slope of the Schild regression was equal to ∼0.5, which may suggest that, although DL-AP4 binds to both protomers of the mGlu_7/8_ heterodimer, the competitive interaction between (*S*)-DCPG and DL-AP4 only occurs through the mGlu_8_ promoter. When performing parallel control Schild regression experiments for mGlu_7/7_ homodimers, (*S*)-DCPG showed no antagonist effect at this receptor (Fig. 3C), as indicated by the lack of correlation between the concentration of (*S*)-DCPG and DR (Fig. 3D, simple linear regression analysis; *r*^2^ = 0.1029, *P* = .483; slope equation: Y = –0.0041x + 4.885). Thus, the lack of (*S*)-DCPG antagonist activity at mGlu_7/7_ homodimers further suggests that the competitive interaction between (*S*)-DCPG and DL-AP4 occurs specifically through the mGlu_8_ promoter of the heterodimer.

### 3.4 Select group III mGlu receptor PAMs show enhanced ability to left-shift the agonist concentration-response at mGlu_7/8_ heterodimers relative to homodimers

Although mGlu_7_ NAMs exhibit differential pharmacology at mGlu_7/8_ heterodimers,^6^ we have yet to characterize the effect of PAMs at mGlu_7/8_ heterodimers in the calcium elevation assay. Thus, we examined the effects of group III PAMs at mGlu_7/8_ heterodimers relative to homodimers in a fold-shift assay. HEK293 cells stably transfected with mGlu_7_- and mGlu_8_-GB-tailed constructs were pretreated with a single concentration of PAM (10 *μ*M) before a full glutamate CRC was applied, after which the functional activity of these receptors was measured in the calcium elevation assay. For each dimer, the leftward shift in glutamate potency was compared in the presence of PAM or vehicle (0.1% DMSO) and reported as a “fold-shift” ratio, where higher fold-shift values indicate greater PAM efficacy.

**Fig. 4:**
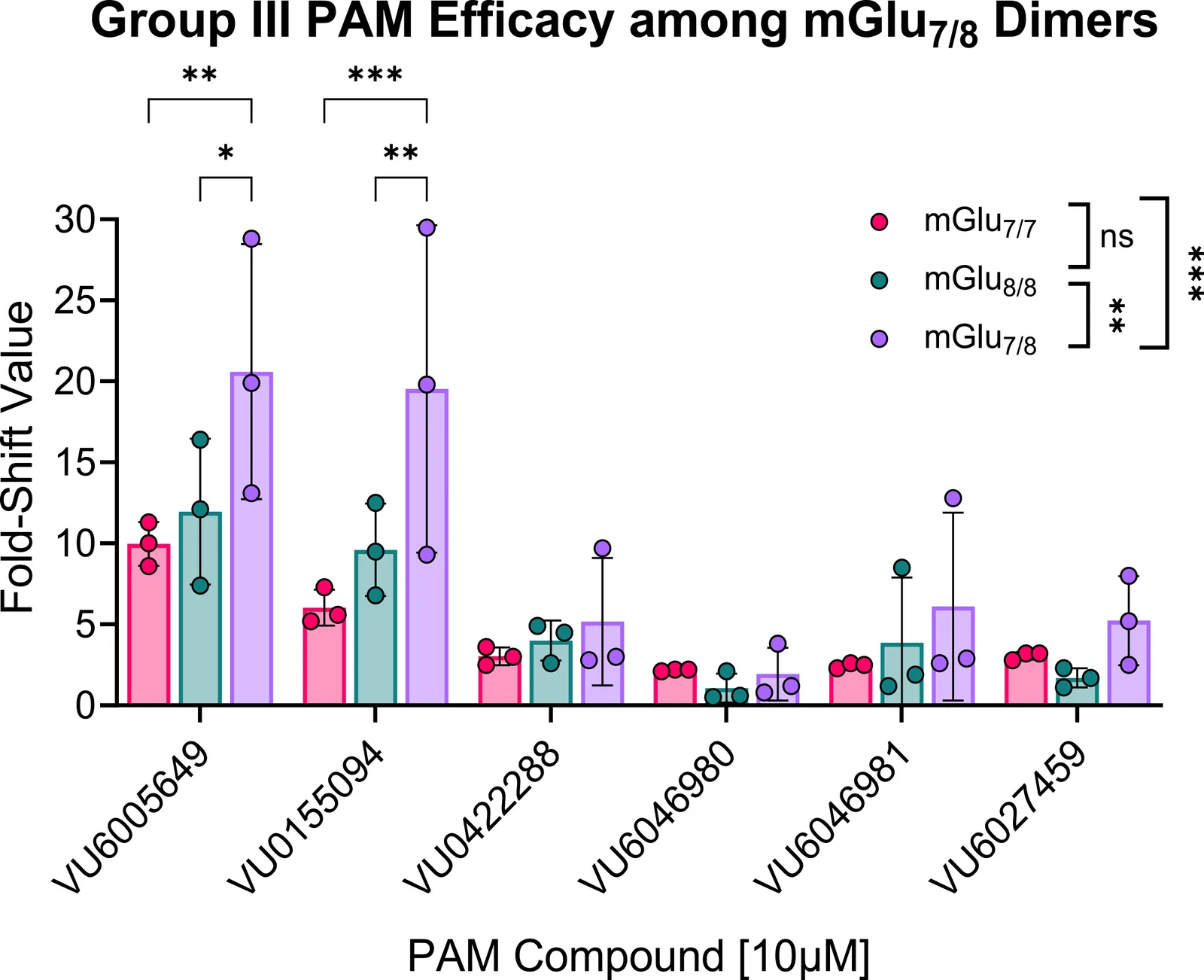
Select group III PAMs show enhanced ability to left-shift the glutamate concentration-response at mGlu_7/8_ heterodimers relative to homodimers. HEK293 cells expressing GB-tailed mGlu_7/8_ dimer constructs were pretreated with a group III PAM before applying increasing concentrations of glutamate. For each dimer, the leftward shift in glutamate potency was compared in the presence of 10 *μ*M PAM or vehicle (0.1% DMSO) and reported as a fold-shift ratio, where higher fold-shift values indicate greater PAM efficacy. Comparing multiple group III PAMs revealed that only VU6005649 and VU0155094 show enhanced efficacy at mGlu_7/8_ heterodimers (2-way ANOVA; main effect of PAM, *P* < .0001; main effect of dimer condition, *P* = .0005; no interaction effect of dimer condition and PAM, *P* = .1465). Post hoc analyses revealed that PAMs showed higher efficacy at mGlu_7/8_ heterodimers relative to mGlu_7/7_ homodimers ∗∗∗*P* < .001 and mGlu_8/8_ homodimers ∗∗*P* < .01, with no significant differences between homodimer conditions *P* = .7648 (ns). For specific PAMs: VU6005649: mGlu_7/7_ vs mGlu_7/8_ ∗∗*P* < .01, mGlu_8/8_ vs mGlu_7/8_ ∗*P* < .05, mGlu_7/7_ vs mGlu_8/8_*P* = .806 (ns). VU0155094: mGlu_7/7_ vs mGlu_7/8_ ∗∗∗*P* < .001, mGlu_8/8_ vs mGlu_7/8_ ∗∗*P* < .01, mGlu_7/7_ vs mGlu_8/8_*P* = .509 (ns). CRCs for individual compounds/dimer conditions are shown in Supplemental Fig. 3. CRC, concentration-response curve; HEK293A, human embryonic kidney 293A; mGlu, metabotropic glutamate receptors; ns, nonsignificant; PAM, positive allosteric modulator.

Our group has discovered and characterized several group III mGlu receptor and mGlu_7_/mGlu_8_ PAMs at various mGlu receptor homodimers.^40^^,^^46^, ^47^, ^48^ Here, we investigated the activity of several of these pharmacological tools—VU0155094, VU6005649, VU0422288, VU6027459, VU6046980, and VU6046981—for PAM activity at mGlu_7_- and mGlu_8_-containing receptors. Glutamate CRCs in response to vehicle and each of the PAMs tested among all 3 dimer conditions are shown in Supplemental Fig. 3. We found that VU0155094 and VU6005649 exhibited significantly higher efficacy in shifting glutamate potency at mGlu_7/8_ heterodimers relative to homodimers (Fig. 4). However, the group III mGlu receptor PAM VU0422288, the mGlu_7_- and mGlu_8_-selective PAM VU6046981, and the mGlu_7_-selective PAMs VU6046980 and VU6027459 did not show the enhanced efficacy of glutamate at the heterodimer relative to homodimers (Fig. 4, 2-way ANOVA; main effect of PAM, *F*(5,36) = 15.43, *P* < .0001; main effect of dimer condition, *F*(2,36) = 9.513, *P* = .0005; no interaction effect of dimer condition × PAM, *F*(10,36) = 1.599, *P* = .1465). Tukey’s post hoc analyses for specific PAMs: VU6005649: mGlu_7/7_ vs mGlu_7/8_ ∗∗*P* < .01, mGlu_8/8_ vs mGlu_7/8_ ∗*P* < .05, mGlu_7/7_ vs mGlu_8/8_ *P* = .806 (nonsignificant [ns]); VU0155094: mGlu_7/7_ vs mGlu_7/8_ ∗∗∗*P* < .001, mGlu_8/8_ vs mGlu_7/8_ ∗∗*P* < .01, mGlu_7/7_ vs mGlu_8/8_ *P* = .509 (ns). For VU0422288, VU6046981, VU6046980, and VU6027459, results were ns among all dimer condition comparisons. The differential effect of various PAMs may be due to the higher general efficacy of VU0155094 and VU6005649 on mGlu_7/7_ and mGlu_8/8_ homodimers, compared with the other PAMs, as shown in Fig. 4. Alternatively, VU0155094 and VU6005649 may bind differently within the heterodimer relative to other mGlu_7_/mGlu_8_ PAMs, which may be why these PAMs show greater efficacy than the other PAMs tested.

### 3.5 Comparison of VU0155094 responses in calcium elevation and GIRK assays reveals differential effects on efficacy

**Fig. 5:**
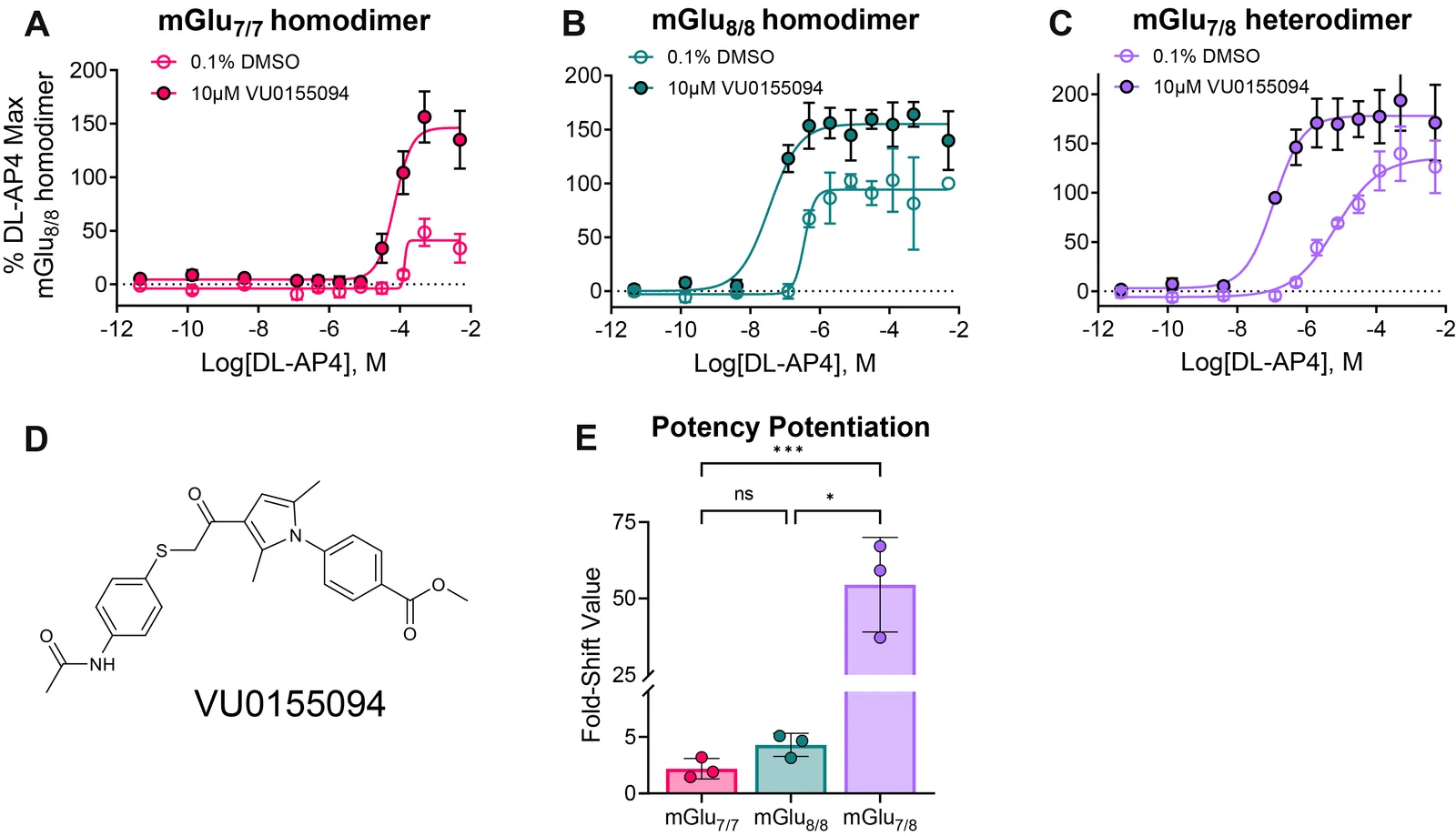
VU0155094 shows robust efficacy to left-shift the agonist concentration-response at mGlu_7/8_ heterodimers in a calcium elevation assay (A). For each dimer condition, 11-point DL-AP4 CRCs were generated to demonstrate how pretreatment with VU0155094 (10 *μ*M) shifts the DL-AP4 potency for the (A) mGlu_7/7_ homodimer (red), (B) mGlu_8/8_ homodimer (teal), and (C) mGlu_7/8_ heterodimers (purple). (D) Chemical structure of the group III PAM VU0155094. (E) The VU0155094-induced leftward shift in DL-AP4 potency was quantified for each dimer condition. Fold-shift values reveal that mGlu_7/8_ heterodimers exhibit a significantly more robust leftward shift in DL-AP4 potency relative to mGlu_7/7_ and mGlu_8/8_ homodimers (1-way ANOVA; *P* = .0006; Tukey’s post hoc mGlu_7/7_ vs mGlu_8/8_*P* = .9558 (ns), mGlu_7/7_ vs mGlu_7/8_ ∗∗∗*P* < .001, and mGlu_8/8_ vs mGlu_7/8_ ∗∗*P* < .01). CRC, concentration-response curve; DL-AP4, DL-2-amino-4-phosphonobutyric acid; mGlu, metabotropic glutamate receptors; ns, ns, nonsignificant; PAM, positive allosteric modulator.

Promiscuous or chimeric G proteins are often used with G_i/o_-coupled GPCRs to induce calcium elevation, which is an efficient kinetic assay that can be used for compound profiling.^56^^,^^59^^,^^60^ To confirm that the G_α15_ protein accurately assesses activation of the conical G_i/o_-coupled activation mechanisms, we profiled the activity of one of the PAMs that showed differential activity at mGlu_7/8_ heterodimers: VU0155094.^46^ After transient expression of mGlu-GB-tailed constructs in HEK293A cells, we generated DL-AP4 and glutamate CRCs in the presence of vehicle or 10 *μ*M VU0155094 using the promiscuous G protein G_α15_ for calcium elevation, or a GIRK-mediated thallium flux experiment mediated by native G_i/o_ proteins. In the calcium elevation assay, we found that VU0155094 enhanced DL-AP4 and glutamate potency at mGlu_7/8_ heterodimers to a greater extent compared with the mGlu_7/7_ and mGlu_8/8_ homodimer conditions (Fig. 5, Supplemental Fig. 4), results similar to our findings in Fig. 4. When using DL-AP4 as the agonist, VU0155094 showed an enhanced ability to left-shift the DL-AP4 response at the mGlu_7/8_ heterodimer (54.5 ± 15.4, fold-shift value ± SD, Fig. 5C) relative to mGlu_7/7_ homodimers (2.2 ± 0.91, Fig. 5A) and mGlu_8/8_ homodimers (4.3 ± 1.0, Fig. 5B) (ordinary 1-way ANOVA; *F*(2,6) = 32.62; *P* = .0006; Tukey’s post hoc mGlu_7/7_ vs mGlu_8/8_ *P* = .9558, mGlu_7/7_ vs mGlu_7/8_ ∗∗∗*P* < .001, and mGlu_8/8_ vs mGlu_7/8_ ∗∗*P* < .01, shown graphically as left-fold-shift, Fig. 5E). When using glutamate as the agonist, similar results were observed (Supplemental Fig. 4). Overall, these data indicate that the group III mGlu receptor PAM VU0155094 exhibits enhanced efficacy in its ability to left-shift agonist potency at mGlu_7/8_ heterodimers relative to homodimers in the calcium elevation assay.

**Fig. 6:**
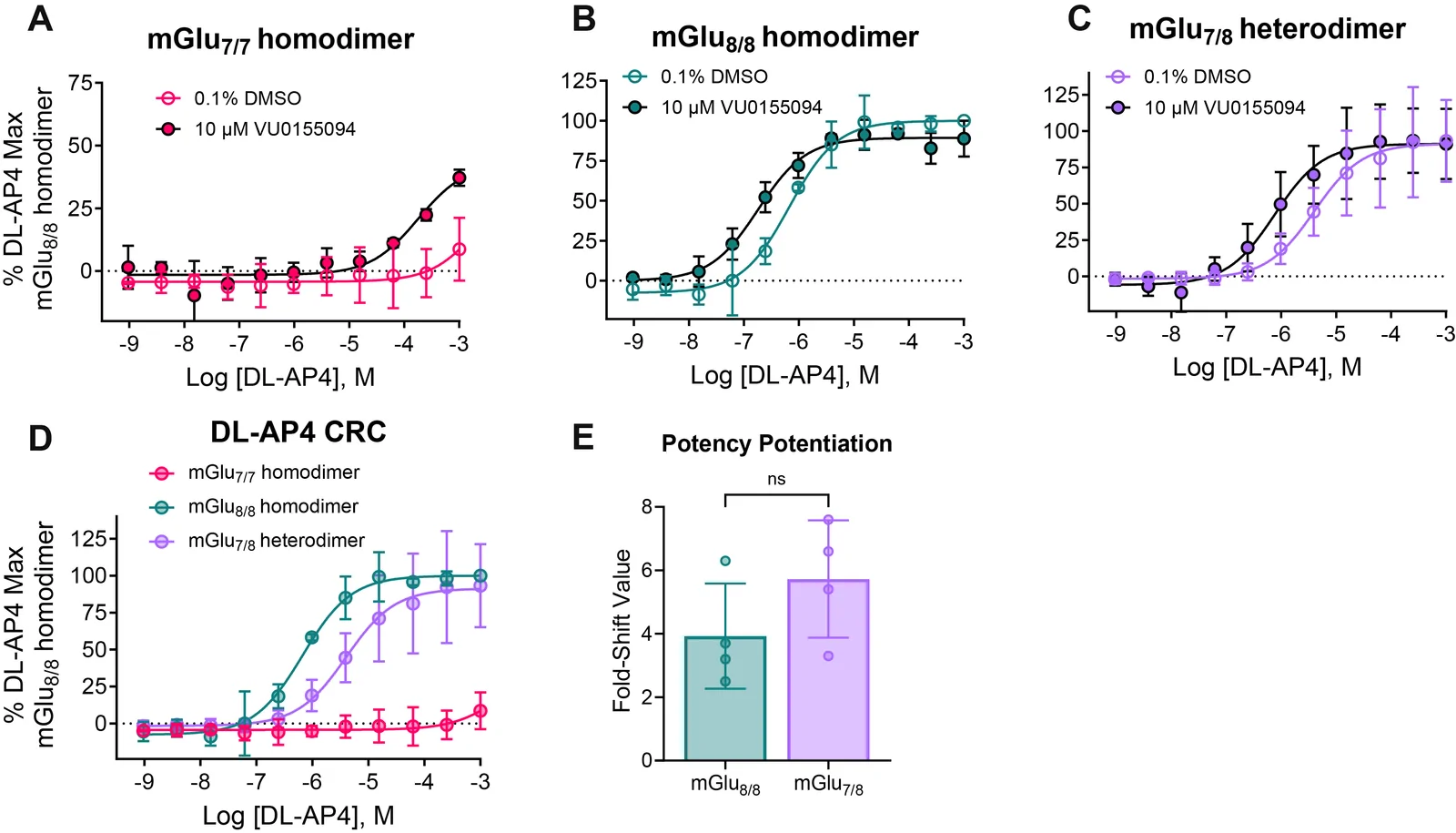
VU0155094 does not show enhanced efficacy at mGlu_7/8_ heterodimers relative to mGlu_8/8_ homodimers in a GIRK-mediated thallium flux assay. Fold-shift comparison of DL-AP4 CRCs showing vehicle (0.1% DMSO) and 10 *μ*M VU0155094 conditions for (A) mGlu_7/7_ homodimers, (B) mGlu_8/8_ homodimers, and (C) mGlu_7/8_ heterodimers in the GIRK-mediated thallium flux assay. (D) DL-AP4 CRCs among mGlu_7/7_ homodimers, mGlu_8/8_ homodimers, and mGlu_7/8_ heterodimers. For mGlu_7/7_ homodimers, the DL-AP4 potency value was not calculated because a curve could not be fit. For mGlu_8/8_ and mGlu_7/8_, the pEC_50_s of DL-AP4 were 6.18 ± 0.06 (mGlu_8/8_) and 5.38 ± 0.14, respectively; (mGlu_7/8_) (mean pEC_50_ ± SD). (E) After analyzing the fold-shift values in the GIRK assay, VU0155094 did not show enhanced efficacy at mGlu_7/8_ heterodimers relative to mGlu_8/8_ homodimers (unpaired *t* test; *t*(6) = 1.1449, *P* = 0.1975 (ns)). CRC, concentration-response curve; DL-AP4, DL-2-amino-4-phosphonobutyric acid; GIRK, G protein–coupled inwardly rectifying potassium channel; mGlu, metabotropic glutamate receptors; ns, ns, nonsignificant.

We next profiled VU0155094 activity using a native G_i/o_-induced GIRK-mediated thallium flux method to both confirm activity of DL-AP4 and glutamate in a G_i/o_ assay at mGlu_7/8_ heterodimers and test the hypothesis that the differential efficacy of VU0155094 observed in the calcium assay would also be observed in an orthogonal assay.^54^^,^^61^^,^^62^ Although DL-AP4 responses at mGlu_7/7_ homodimers showed minimal efficacy relative to mGlu_8/8_ homodimers at the highest concentration tested (1 mM) in this assay, VU0155094 was able to potentiate the DL-AP4 response at mGlu_7/7_ homodimers (Fig. 6A). As a best-fit could not be calculated for the DL-AP4 vehicle (0.1% DMSO) CRC at mGlu_7/7_ homodimers, potency and fold-shift values were not calculated for this condition. When testing DL-AP4 and VU0155094 for efficacy at mGlu_8/8_ homodimers (Fig. 6B) and mGlu_7/8_ heterodimers (Fig. 6C), VU0155094 showed consistent PAM effects at both receptors. When comparing DL-AP4 CRCs in the absence of this PAM in the GIRK assay, each of the 3 dimers tested shows similar responses to those seen in the calcium elevation assay (Fig. 6D). DL-AP4 showed greater potency at mGlu_8/8_ homodimers (6.18 ± 0.06; mean pEC_50_ ± SD) relative to mGlu_7/8_ heterodimers (5.38 ± 0.14; mean pEC_50_ ± SD, unpaired *t* test; *t*(6) = 10.29; ∗∗∗∗*P* < .0001). For glutamate, the pEC_50_ potency was 4.62 ± 0.19 at mGlu_8/8_ homodimers and 4.09 ± 0.16 for mGlu_7/8_ heterodimers (Supplemental Fig. 5B), with glutamate being more potent at mGlu_8/8_ homodimers compared with mGlu_7/8_ heterodimers (unpaired *t* test; *t*(6) = 4.225; ∗∗*P* < .01). The potency of DL-AP4 and glutamate among all 3 dimers tested in the calcium assay and GIRK assay are summarized in Table 1. No differences were detected when comparing fold-shift values between mGlu_8/8_ homodimers (3.9 ± 1.7) and mGlu_7/8_ heterodimers (5.7 ± 1.9) (unpaired *t* test; *t*(6) = 1.449; *P* = .1975), suggesting that VU0155094 shows enhanced PAM efficacy only in the calcium elevation assay using a promiscuous G protein (Fig. 6E). We also examined the efficacy of VU0155094 in response to glutamate (Supplemental Fig. 5), and these experiments yielded similar results to those performed when using DL-AP4 as the agonist. Despite the lack of response from mGlu_7/7_ homodimers to glutamate alone (0.1% DMSO), in the presence of 10 *μ*M VU0155094, mGlu_7/7_ homodimers elicited a functional response at high concentrations of glutamate (Supplemental Fig. 5B), indicating PAM activity of this compound at mGlu_7/7_ homodimers as previously reported.^46^ We also observed leftward shifts in glutamate potency induced by VU0155094 at mGlu_8/8_ homodimers (Supplemental Fig. 5C) and mGlu_7/8_ heterodimers (Supplemental Fig. 5D). When comparing glutamate fold-shift values between mGlu_8/8_ homodimers and mGlu_7/8_ heterodimers, no differences were detected (unpaired *t* test; *t*(6) = 1.597, *P* = .1613, Supplemental Fig. 5E). These results indicate that the effects on differential potentiation of VU0155094 are limited to the promiscuous G protein assay. Importantly, however, they confirm that this compound can potentiate activity of both agonists at mGlu_7/8_ heterodimers.

**Table 1:** DL-AP4 and glutamate exhibit differential potencies at mGlu_7/7_, mGlu_8/8_, and mGlu_7/8_ Summary of agonist pEC_50_ at each receptor tested (mGlu_7/7_ homodimers, mGlu_8/8_ homodimers, and mGlu_7/8_ heterodimers) in calcium elevation and GIRK thallium flux assays. Potencies are denoted as pEC_50_ ± SD (*N* = 3–4 independent experiments).

	Calcium Assay		GIRK Assay	
	DL-AP4	Glutamate	DL-AP4	Glutamate
mGlu_7/7_	3.86 ± 0.01	N/A	N/A	N/A
mGlu_8/8_	6.41 ± 0.06	5.73 ± 0.10	6.18 ± 0.06	4.62 ± 0.19
mGlu_7/8_	5.17 ± 0.20	5.05 ± 0.14	5.38 ± 0.14	4.09 ± 0.16

DL-AP4, DL-2-amino-4-phosphonobutyric acid; GIRK, G protein–coupled inwardly rectifying potassium channel; mGlu, metabotropic glutamate receptors; N/A, not applicable.

### 3.6 (S)-DCPG lacks agonist activity at mGlu_7/8_ heterodimers in the modulation of GIRK channels and exhibits antagonist activity at mGlu_7/8_ heterodimers in a CODA-RET assay

**Fig. 7:**
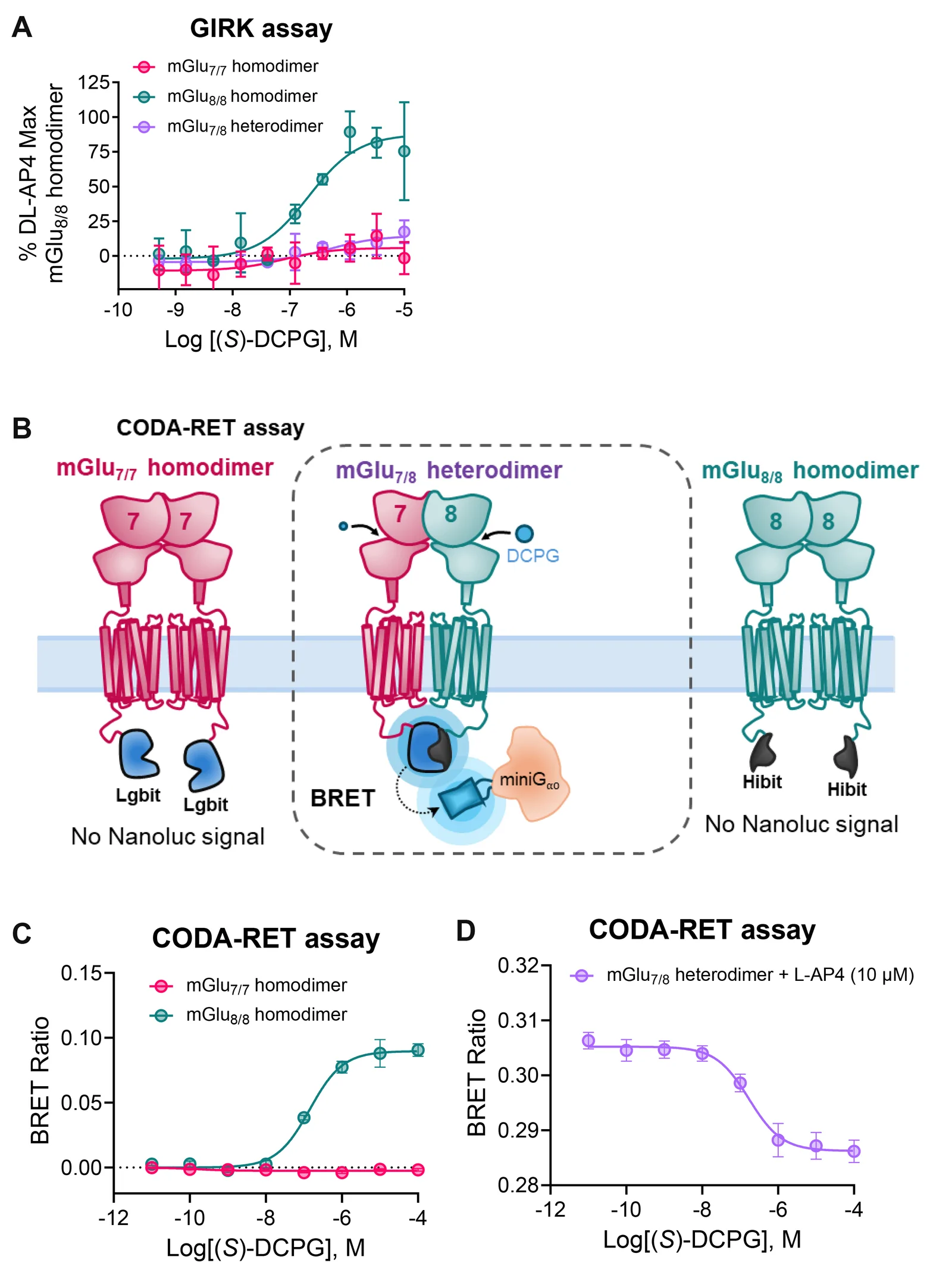
(*S*)-DCPG maintains reduced activity in a GIRK-mediated thallium flux assay and antagonist activity in a complemented donor acceptor resonance energy transfer (CODA-RET) assay. (A) CRCs in response to the mGlu_8_ agonist (*S*)-DCPG shown for mGlu_7/7_ homodimers (red), mGlu_8/8_ homodimers (teal), and mGlu_7/8_ heterodimers (purple) reveals that, in contrast to effects at mGlu_8/8_ homodimers, (*S*)-DCPG is a very weak agonist at mGlu_7/8_ heterodimers (paired *t* test for (*S*)-DCPG baseline vs maximum efficacy at mGlu_7/8_ heterodimers; *t*(2) = 5.425, ∗*P* < .05). (B) A CODA-RET assay was used to selectively measure G protein recruitment by mGlu_7/8_ heterodimers without contribution of signaling from homodimers expressed in the same cells. (C) In CODA-RET, (*S*)-DCPG is an agonist at mGlu_8/8_ homodimers and shows no activity at mGlu_7/7_ homodimers. (D) In the presence of an EC_80_ concentration of L-AP4, (*S*)-DCPG shows dose-dependent antagonist activity at mGlu_7/8_ heterodimers (pIC_50_ = 6.76 ± 0.17). BRET, bioluminescence resonance energy transfer; CRC, concentration-response curve; DL-AP4, DL-2-amino-4-phosphonobutyric acid; GIRK, G protein–coupled inwardly rectifying potassium channel; mGlu, metabotropic glutamate receptors; (*S*)-DCPG, (*S*)-3,4-dicarboxyphenylglycine.

Based on our findings that (*S*)-DCPG did not activate mGlu_7/8_ heterodimers in the calcium elevation assay, as well as the assay-dependent differential effects of VU0155094 PAM pharmacology, we profiled the activity of (*S*)-DCPG in the GIRK thallium flux assay to compare activity between the 2 assays. We generated (*S*)-DCPG CRCs and normalized GIRK functional activity to a percent maximal DL-AP4 activation at mGlu_8/8_ homodimers (Fig. 7A). Similar to our observations in the calcium assay, (*S*)-DCPG showed robust functional activity at mGlu_8/8_ homodimers (88.4% ± 17.9%); in contrast, (*S*)-DCPG showed no functional activity mGlu_7/7_ homodimers (1.0% ± 9.2%) and extremely weak partial agonist activity at mGlu_7/8_ heterodimers (16.6% ± 9.2%, data normalized to percent maximal DL-AP4 activation [1 mM] at mGlu_8/8_ homodimers; mean ± SD; ordinary 1-way ANOVA; *F*(2,6) = 40.07, ∗∗∗*P* < .001; Tukey’s post hoc for individual dimers: mGlu_7/7_ vs mGlu_8/8_ ∗∗∗*P* < .001; mGlu_7/7_ vs mGlu_7/8_ *P* = .3575, mGlu_8/8_ vs mGlu_7/8_ ∗∗*P* < .01). The very weak agonist activity of (*S*)-DCPG at mGlu_7/8_ heterodimers observed in the GIRK assay, but not calcium elevation, may be due to the greater sensitivity of the GIRK assay to detect “leak” of mGlu_8/8_ homodimers from the GB tails retention system relative to the calcium assay due to native G_i/o_ coupling. These findings suggest that (*S*)-DCPG is an mGlu_8/8_ homodimer-selective agonist relative to mGlu_7/7_ homodimers and mGlu_7/8_ heterodimers in the native G_i/o_ GIRK assay.

We also examined the activity of (*S*)-DCPG at mGlu_7/8_ heterodimers in a CODA-RET assay, which is used to selectively measure signaling by mGlu_7/8_ heterodimers without contribution of signaling from homodimers expressed in the same cells^6^^,^^44^^,^^45^ (Fig. 7B). Similar to our observations in the calcium elevation and GIRK assays with mGlu-GB-tailed heterodimer constructs, in CODA-RET, (*S*)-DCPG showed concentration-dependent agonist activity at mGlu_8/8_ homodimers and no agonist activity at mGlu_7/7_ homodimers (Fig. 7C). When applying an EC_80_ concentration of L-AP4 (10 *μ*M) to activate mGlu_7/8_ heterodimers in the presence of an (*S*)-DCPG CRC, we observed dose-dependent inhibition of mGlu_7/8_ heterodimer activity with a pIC_50_ of 6.76 ± 0.17 (pIC_50_ ± SD) for (*S*)-DCPG (Fig. 7D). These findings confirm that (*S*)-DCPG acts as an antagonist at mGlu_7/8_ heterodimers, demonstrating that this compound displays unique mode-switching pharmacology at mGlu_7/8_ heterodimers relative to mGlu_8/8_ homodimers.

Overall, these data suggest that (*S*)-DCPG is a selective full agonist of mGlu_8/8_ homodimers and exhibits a strong preference for mGlu_8/8_ versus either mGlu_7/7_ homodimers or mGlu_7/8_ heterodimers. These results also indicate that (*S*)-DCPG could be used as a tool to potentially identify mGlu_7/8_ dimers in native tissues due to the ability of this compound to still bind to the mGlu_7/8_ heterodimer and block the effects of agonists (Fig. 3).

## 4 Discussion

The current study demonstrates that the mGlu_7/8_ heterodimer displays unique pharmacology relative to mGlu_7/7_ and mGlu_8/8_ homodimers and that all 3 of these receptors possess distinct selectivity profiles in response to orthosteric agonists and allosteric modulators. Specifically, we show that the mGlu_7/8_ heterodimer possess 2 nonequivalent orthosteric binding sites for the same ligand and likely requires both agonist binding sites to be occupied for saturable activation of the receptor. This interpretation is based on the observations that (1) the mGlu_7/8_ heterodimer exhibits a significantly shallower Hill slope compared with homodimers and (2) the selective mGlu_8_ agonist (*S*)-DCPG is unable to effectively activate the mGlu_7/8_ heterodimer in 3 assays. The finding that (*S*)-DCPG is a selective full agonist for mGlu_8/8_ homodimers over mGlu_7/8_ heterodimers is crucial for our pharmacological and biological understanding of mGlu_8_, as our laboratory and others have shown that (*S*)-DCPG is pharmacologically active at SC-CA1 synapses in the hippocampus of neonatal rodents but not adults.^63^^,^^64^ Therefore, it is plausible that, while mGlu_8/8_ homodimers exist in the hippocampus during early development,^63^^,^^64^ mGlu_8_ may be more likely to exist in heterodimer conformations in the hippocampus during adulthood. In support of this hypothesis, we have previously demonstrated that mGlu_7/8_ heterodimers may regulate the induction of synaptic plasticity at SC-CA1 synapses in adult mice.^6^ However, it remains unclear whether (*S*)-DCPG is a selective mGlu_8/8_ homodimer agonist relative to all mGlu_8_-containing heterodimer confirmations, as mGlu heterodimers have been shown to exhibit distinct pharmacological profiles relative to mGlu homodimers and other heterodimers.^45^^,^^65^^,^^66^ Therefore, additional studies investigating the biological role of mGlu_7/8_ heterodimers in the hippocampus using *Grm8* KO mice are needed to support the hypothesis that mGlu_8_ is expressed predominately in heterodimer confirmations in the adult hippocampus.

Evidence that (*S*)-DCPG acts as a competitive antagonist at mGlu_7/8_ heterodimers is interesting, especially considering that this compound has been characterized as a highly selective mGlu_8_ agonist.^57^^,^^67^ Although Schild regression analysis implies that the interaction between 2 ligands is only competitive if the slope is equal to 1,^68^^,^^69^ the linear slope of the Schild regression suggests that the interaction between DL-AP4 and (*S*)-DCPG at the mGlu_7/8_ heterodimer is competitive. As the DL-AP4 Hill slope is less than 1 for mGlu_7/8_ heterodimers, the Schild regression slope value of ∼0.5 suggests that the competitive interaction between DL-AP4 and (*S*)-DCPG only occurs at the mGlu_8_ subunit of the dimer. Therefore, (*S*)-DCPG may act as a selective competitive antagonist at the mGlu_7/8_ heterodimer by blocking agonist activity through a single protomer of a heterodimeric receptor that requires 2 agonist binding sites for maximal functional activity. Previous reports suggest that a Schild slope of less than 1 implies the presence of an additional antagonist “removal” site that results in a lower free concentration of antagonist available to compete with agonist.^70^, ^71^, ^72^ However, rather than an antagonist “removal” site, interpretation of our results suggests that the Schild slope relates to the ratio of antagonist binding sites to agonist binding sites on the receptor. In the case of the mGlu_7/8_ heterodimer, the ratio of antagonist to agonist binding sites is 1:2, thus resulting in a Schild slope of 0.5.

Structural evidence has also shown that (*S*)-DCPG does not enable full closure of the mGlu_8_ Venus flytrap domain (VFD), despite the compound showing full agonist activity at the receptor in homodimer confirmation.^73^^,^^74^ Therefore, (*S*)-DCPG may cause a unique confirmational change in mGlu_8/8_ homodimers to induce saturable levels of receptor activity irrespective of full VFD closure. However, when (*S*)-DCPG is bound to the mGlu_8_ protomer in the mGlu_7/8_ heterodimer confirmation, the compound may prevent sufficient reorientation of the VFD in manner that hinders receptor activation, thereby acting as an antagonist.

In our CODA-RET experiments, (*S*)-DCPG displayed similar potency in agonist mode at mGlu_8/8_ homodimers (143 nM) and antagonist mode (170 nM) at mGlu_7/8_ heterodimers. Thus, the presumed affinity of (*S*)-DCPG for the mGlu_8_ protomer may be the same regardless of whether the receptor is in a homodimer or heterodimer confirmation. This suggests that (*S*)-DCPG may induce confirmational changes that are unique to when mGlu_8_ is in a homodimer confirmation as opposed to a heterodimer. Importantly, these findings also suggest that (*S*)-DCPG may be used in native tissues as a competitive antagonist to block mGlu_7/8_ heterodimer-mediated effects of the group III agonist L-AP4, and future studies will address this hypothesis. In addition, use of (*S*)-DCPG in native tissues will require examination of compound activity at all mGlu_8_-containing heterodimers, and these experiments are currently underway.

In the present study, we demonstrated that specific group III PAMs exhibit significantly greater efficacy at the mGlu_7/8_ heterodimer relative to mGlu_7/7_ and mGlu_8/8_ homodimers in the promiscuous G protein assay, and we showed that the group III PAM VU0155094 exhibits an enhanced ability to left-shift responses to DL-AP4 and glutamate in this assay. It has been shown that VU0422288 binds at the 7-transmembrane domain (7TM) interface of mGlu_8/8_ homodimers.^74^ However, it is unknown whether VU0422288 also binds at the 7TM interface of mGlu_7/8_ heterodimers. Unlike VU0155094 and VU6005649, VU0422288 did not show enhanced efficacy at mGlu_7/8_ heterodimers relative to homodimers. Although it is currently unknown where each of the PAMs tested binds to within the 7TM of mGlu_7/8_ heterodimers, it is reasonable to hypothesize that PAMs that show enhanced efficacy at mGlu_7/8_ heterodimers bind differentially within the 7TM interface of the dimer relative to PAMs that do not enhance efficacy at the heterodimer. Alternatively, these findings may be due to overall weaker coupling of mGlu_7_ to promiscuous G proteins compared with the other receptors. Evidence also indicates that mGlu heterodimers exhibit unique molecular dynamics related to enhanced efficacy of the receptor,^66^^,^^75^ which may relate to the differential efficacy of group III agonists and PAMs among mGlu_7/7_, mGlu_8/8_, and mGlu_7/8_ dimer conformations observed here.

When assessing the efficacy of VU0155094 at mGlu_7/8_ heterodimers relative to mGlu_8/8_ homodimers in the GIRK assay, we did not observe greater efficacy of this PAM at the heterodimer in this assay. The difference in PAM efficacy at the heterodimer relative to homodimers in the calcium assay relative to the GIRK assay warrants follow-up studies to test the hypothesis that PAMs show greater efficacy at mGlu_7/8_ heterodimers relative to homodimers in additional functional assays that are reflective of endogenous mGlu receptor signaling cascades. In addition, future studies investigating G protein coupling rates among heterodimers and homodimers would help shed light on the molecular mechanisms by which heterodimers exhibit differential pharmacology relative to homodimers in response to agonists and PAMs. Of the 6 PAMs tested, we have reported 2 as selective for group III mGlu receptors (VU0155094 and VU0422288), 2 selective for both mGlu_7_ and mGlu_8_ (VU6005649 and VU6046981), and 2 selective for mGlu_7_ only (VU6027459 and VU6046980).^40^^,^^46^, ^47^, ^48^ From these results, it appears that the selectivity of these PAMs for mGlu_7_ over other group III receptors does not play a significant role in PAM efficacy at mGlu_7/8_ heterodimers. Although not examined in the present study, future experiments could focus on further profiling potential signaling bias of the mGlu_7/8_ beyond calcium elevation and GIRK assays, such as in cyclic AMP accumulation and β-arrestin recruitment assays,^76^^,^^77^ particularly since mGlu_8_ has recently been shown to exhibit unique *β*-arrestin trafficking dynamics.^74^

Although our group and others have identified several compounds that are selective at the various subtypes of mGlu homodimers, to our knowledge, there has yet to be a ligand identified that selectively modulates an mGlu heterodimer subtype relative to homodimers. Considering the role of mGlu_7/8_ heterodimers in hippocampal neural plasticity^6^ and findings that *Grm7* and *Grm8* KO mice show overlapping associative learning deficits in the hippocampal-dependent memory task contextual fear conditioning,^30^^,^^33^^,^^34^ one hypothesis is that these deficits in contextual hippocampal-dependent associative learning are due to disrupted mGlu_7/8_ heterodimer function in the hippocampus. In line with this hypothesis, VU6005649 exhibits robust PAM efficacy at mGlu_7/8_ heterodimers and enhances contextual fear conditioning associative learning in wild-type mice.^40^ Therefore, further profiling the physiological activity of VU6005649 at SC-CA1 synapses during LTP induction, as well as investigating the behavioral efficacy of this compound in *Grm7* and *Grm8* KO mice, will be crucial for understanding the role of mGlu_7/8_ heterodimers in hippocampal plasticity, learning, and memory.

Because of the recognition of mGlu receptor heterodimers in vivo, selectively targeting mGlu_7/8_ heterodimers with small-molecule allosteric modulators may serve as an effective and highly selective therapeutic strategy for treating neurodevelopmental disorders associated with deficits in hippocampal neural plasticity and associative learning. This strategy may also have the advantage of limiting off-target effects at mGlu_7_- and mGlu_8_-containing receptor homodimers while precisely targeting neural circuits associated with mGlu_7/8_ heterodimer-mediated modulation of neural plasticity. For example, an mGu_7_ NAM compound (ADX71743) we have shown to inhibit mGlu_7/8_ heterodimers and block LTP at SC-CA1 synapses elicits seizures in mice, whereas an mGlu_7_ NAM compound (6-(4-Methoxyphenyl)-5-methyl-3-(4-pyridinyl)-isoxazolo[4,5-c]pyridin-4(5H)-one hydrochloride [MMPIP]) that does not inhibit the heterodimer nor block SC-CA1 LTP does not exacerbate seizure induction.^6^^,^^78^^,^^79^ Thus, compounds that selectively potentiate mGlu_7/8_ heterodimers could also be explored as potential candidate compounds that affect epilepsy-like phenotypes in rodents. These types of studies would assist in determining the ideal receptor conformation that should be targeted for therapeutic effects.

## 5 Conclusion

Overall, the present findings highlight the unique pharmacological properties of mGlu_7/8_ heterodimers, which have been hypothesized to play a role in hippocampal synaptic plasticity.^6^ Importantly, this study provides strong rationale for generating and employing a pharmacological toolkit comprising group III mGlu orthosteric agonists, antagonists, and allosteric modulators to selectively probe and distinguish among mGlu_7/7_, mGlu_8/8_, and mGlu_7/8_ dimers in native tissues and in vivo. In addition, these studies will be critical for determining which of these 3 GPCR dimer receptor populations are responsible for modulating hippocampal neural plasticity and associative learning in mice.^40^ Taken together, the molecular characterization of mGlu_7/8_ heterodimers reveals unique properties of group III agonists and PAMs that contribute to our understanding of group III mGlu receptor pharmacology and biology and establishes a rationale for investigating the therapeutic efficacy of this unique GPCR heterodimer.

## Conflict of interest

Colleen M. Niswender reports financial support from National Institute of Mental Health and National Institute of Neurological Disorders and Stroke. All other authors declare no conflicts of interest.
