# Supplementary material for: Molecular pharmacology of mGlu7/8 heterodimers reveals unique properties of orthosteric agonists and positive allosteric modulators
Source: Mol Pharmacol. 2026 Jun 6;108(7):100133. doi: 10.1016/j.molpha.2026.100133 (PMC13430957; doi:10.1016/j.molpha.2026.100133)
Supplement: Supplementary file 1 [file mmc1.docx]

**Molecular pharmacology of mGlu_7/8_ heterodimers reveals unique properties of orthosteric agonists and positive allosteric modulators**

Anthony S. Ferranti^a,b^, Youhyun Noh^a,b^, Xia Lei^a,b^, Xin Lin^f,g^, Joseph D. Bungard^a,b^, Wesley B. Asher^f,g^, Jonathan A. Javitch^f,g^, Colleen M. Niswender^†, a,b,c,d,e^

a. Department of Pharmacology, Vanderbilt University, Nashville, TN, 37232, USA
b. Warren Center for Neuroscience Drug Discovery and Vanderbilt Institute for Translational Advances, Nashville, TN, 37232, USA
c. Vanderbilt Kennedy Center, Vanderbilt University Medical Center, Nashville, TN, 37232, USA
d. Vanderbilt Brain Institute, Vanderbilt University, Nashville, TN, 37232, USA
e. Vanderbilt Institute of Chemical Biology, Vanderbilt University, Nashville, TN, 37232, USA
f. Departments of Psychiatry and Molecular Pharmacology and Therapeutics, Columbia University Vagelos College of Physicians * Surgeons, New York, NY 10032, USA
g. Division of Molecular Therapeutics, New York State Psychiatric Institute, New York, NY 10032, USA

^†^Correspondence to:

Colleen M. Niswender, Ph.D.

Associate Professor of Pharmacology

Warren Center for Neuroscience Drug Discovery

1215D Light Hall

Vanderbilt University

Nashville, TN 37232-0697

Tel. (615) 343-4303

e-mail: Colleen.niswender@vanderbilt.edu

Article Type: Research Manuscript

Journal: *Molecular Pharmacology*

Special Issue: *Emergent Pharmacology of Allosteric GPCR Ligands*

Keywords: Allosteric Modulators, G protein-coupled receptors, mGlu, CNS disorders

**Supplemental Figure 1: No significant differences between heterodimer configurations following transient transfections at efficacious concentrations of agonist in the calcium elevation assay.** To functionally characterize the molecular pharmacology of mGlu_7/8_ heterodimers in HEK293 cells, two separate transfections were performed for each mGlu_7/8_ heterodimer configuration: 1) mGlu_8_ GB1 + mGlu_7_ GB2 (denoted as mGlu_8/7_ heterodimer; light purple), or 2) mGlu_7_ GB1 + mGlu_8_ GB2 (denoted as mGlu_7/8_ heterodimer; dark purple). In all experiments, each separate heterodimer configuration was run on the same plate to assess potential differences. During post-hoc analysis, heterodimer responses were combined. (**A**) No significant differences in maximal calcium response were detected between each heterodimer in response to glutamate (two-way ANOVA; no main effect of heterodimer configuration F(1, 24) = 3.181, p=0.0871 (ns); main effect of concentration F(5, 24) = 8.665, p<0.0001; no interaction effect F(5, 24) = 0.1114, p=0.9887). (**B**) No significant differences in glutamate potency between heterodimers were detected (unpaired t-test; t(4) = 0.4422, p=0.6812 (ns)). (**C**) Comparison between each heterodimer in response to the agonist DL-AP4 revealed no significant differences (two-way ANOVA; no main effect of heterodimer configuration F(1, 28) = 0.2082, p=0.6517 (ns); main effect of concentration F(6, 28) = 16.74, p<0.0001; no interaction effect F(6, 28) = 0.07543, p=0.07543). (**D**) No significant differences in DL-AP4 potency between heterodimers were detected (unpaired t-test; t(4) = 0.4770, p=0.6583 (ns)). *N*=3; averaged triplicates obtained on separate days. Data plotted are mean ± SD.

**A**

**B**

**C**

**D**

**B**

**A**

**Supplemental Figure 2: Transient transfections with mGlu GB-tailed control conditions reveals that only the mGlu_8_ GB2 condition shows functional leak in response.** Control conditions were generated by transfecting one mGlu GB-tailed construct with empty vector (pcDNA3), or two mGlu GB-tailed constructs containing the same GB tail (e.g., mGlu_7_ GB1 + mGlu_8_ GB1) and were tested to detect for functional “leak” from the various mGlu GB-tailed constructs in the calcium elevation assay. (**A**) The mGlu_8_ GB2 control condition (pcDNA3 + mGlu_8_ GB2) shows functional leak (19.6% max efficacy) relative to the mGlu_8/8_ homodimer condition (mGlu_8_ GB1 + mGlu_8_ GB2) at 100% efficacy. *N*=3. Conditions run in triplicate. Data plotted are mean ± SD. (**B**) Conditions transfected only with the mGlu_8_ GB2 construct display low levels of DL-AP4 agonist-induced efficacy activity relative to dimer conditions, whereas mGlu_7_ GB2, mGlu_7_ GB1, or mGlu_8_ GB1 control conditions do not reveal detectable levels of functional leak. *N*=1. Conditions run in triplicate. Data plotted are mean ± SD.

**Supplemental Figure 3. Glutamate fold-shift CRCs at each dimer condition for the various PAMs tested.** (**A-F**) mGlu_7/7_ homodimer CRCs for all 6 PAMs tested in glutamate fold-shift assay in calcium elevation assay. (**G-L**) mGlu_8/8_ homodimer CRCs, and (**M-R**) mGlu_7/8_ heterodimer CRCs for battery of 6 PAMs tested. For each dimer condition, data are normalized to percent max glutamate activation for the vehicle (DMSO) CRC within each respective dimer condition (i.e. mGlu_7/7_ homodimer glutamate (vehicle) CRC = 100%; mGlu_8/8_ homodimer glutamate (vehicle) CRC = 100%; mGlu_7/8_ heterodimer glutamate (vehicle) CRC = 100%. *N*=3 independent experiments; averaged duplicates; mean ± SD.

**A**

**B**

**C**

**D**

**E**

**F**

**G**

**H**

**I**

**J**

**K**

**L**

**M**

**N**

**O**

**P**

**Q**

**R**

**Supplemental Figure 4: VU0155094 shows enhanced potentiation of mGlu_7/8_ heterodimers in response to glutamate compared to homodimers** (**A**). An 11-point glutamate CRC demonstrates how pretreatment with a single concentration of VU0155094 (10 µM) shifts the glutamate potency for the (A) mGlu_7/7_ homodimer (red), (**B**) mGlu_8/8_ homodimer (teal), and (**C**) mGlu_7/8_ heterodimer (purple). *N*=3; triplicates obtained on 3 separate days, mean ± SD. (**D**) The VU0155094-induced leftward shift in glutamate potency was quantified for each dimer condition. Fold-shift values revealed that mGlu_7/8_ heterodimers exhibited a significantly more robust leftward shift in glutamate potency following pretreatment with 10 µM VU0155094 relative to mGlu_7/7_ and mGlu_8/8_ homodimers. Ordinary one-way ANOVA, F(2, 6) = 17.11, p<0.01 (**) main effect of dimer condition; Tukey’s post hoc mGlu_7/7_ vs. mGlu_8/8_ p=0.5914 (ns), mGlu_7/7_ vs. mGlu_7/8_ p<0.01 (**), and mGlu_8/8_ vs. mGlu_7/8_ p=0.01 (**).

**D**

**C**

**B**

**A**

**C**

**B**

**E**

**D**

**A**

**Supplemental Figure 5. VU0155094 does not show enhanced glutamate fold-shift efficacy at mGlu_7/8_ heterodimers relative to mGlu_8/8_ homodimers in the GIRK assay.** (**A**) Comparison of glutamate CRCs (vehicle only) for mGlu_7/7_ homodimers (red), mGlu_8/8_ homodimers (teal), and mGlu_7/8_ heterodimers (purple) after transient expression. The glutamate pEC_50_ was 4.62 ± 0.19 at mGlu_8/8_ homodimers and 4.09 ± 0.16 for mGlu_7/8_ heterodimers (mean pEC_50_ ± SD). For mGlu_7/7_ homodimers, no functional efficacy was observed with glutamate alone. Glutamate CRCs in GIRK thallium flux assay and fold-shift comparisons showing vehicle (0.1% DMSO) and 10 µM VU0155094 conditions for (**B**) mGlu_7/7_ homodimers, (**C**) mGlu_8/8_ homodimers, (**D**) and mGlu_7/8_ heterodimers. (**E**) In the GIRK fold-shift assay, VU0155094 did not show enhanced efficacy at mGlu_7/8_ heterodimers relative to mGlu_8/8_ homodimers (unpaired t-test; t(6) = 1.597, p=0.1613 (ns)).
